# BIO 300 Attenuates Whole Blood Transcriptome Changes in Mice Exposed to Total-Body Radiation

**DOI:** 10.3390/ijms25168818

**Published:** 2024-08-13

**Authors:** Artur A. Serebrenik, Oluseyi O. Fatanmi, Stephen Y. Wise, Sarah A. Petrus, Michael D. Kaytor, Vijay K. Singh

**Affiliations:** 1 Humanetics Corporation, Minneapolis, MN 55435, USA; 2 Division of Radioprotectants, Department of Pharmacology and Molecular Therapeutics, F. Edward Hébert School of Medicine, Uniformed Services University of the Health Sciences, Bethesda, MD 20814, USA; oluseyi.fatanmi@usuhs.edu (O.O.F.);; 3 Armed Forces Radiobiology Research Institute, Uniformed Services University of the Health Sciences, Bethesda, MD 20814, USA

**Keywords:** BIO 300, countermeasure, irradiation, mice, biomarker, transcriptome

## Abstract

Development of radiation medical countermeasures under the U.S. Food and Drug Administration Animal Rule requires the capability to translate an effective animal-to-human drug dose. One method of human dose translation is using a biomarker and determining drug doses that modulate the biomarker to the desired level. BIO 300 Oral Powder (BIO 300) is a prophylactic radiation medical countermeasure that is currently being developed following the Animal Rule. The present study aimed to identify biomarkers that can be used for human dose conversion by conducting transcriptomics of whole blood collected from BIO 300-treated CD2F1 mice in the presence and absence of total-body irradiation (TBI). Unirradiated mice were treated with vehicle or 50, 100, or 200 mg/kg BIO 300, and irradiated mice were treated with 200 mg/kg or BIO 300 or vehicle prior to TBI. Whole-blood samples were collected after the last dose of the drug and after irradiation. RNA sequencing demonstrated 100 and 200 mg/kg of BIO 300 doses caused significantly more differential gene expression at 48 h after drug dose compared to 50 mg/kg of BIO 300 (7648, 7680, and 55 significantly differently expressed genes, respectively). Interestingly, following TBI, there were no significantly differentially expressed genes between vehicle- and BIO 300-treated mice. Despite the lack of significant changes in gene expression, the transcriptomic profiles in both groups indicated differential changes in signaling pathways. Pathway analysis of the transcriptome profile from vehicle-treated/TBI mice revealed that many inflammatory signaling pathways were activated in these animals. Signaling pathways enriched in BIO 300-treated/TBI mice were involved in cellular stress and immune response and were predicted to be inhibited. In all, four signaling pathways of interest were identified that were differentially enriched in irradiated animals treated with BIO 300: pathogen-induced cytokine storm signaling, S100 family signaling, pulmonary fibrosis idiopathic signaling, and wound-healing signaling. These pathways should be explored to identify potential biomarkers of BIO 300 that can be used for human dose translation.

## 1. Introduction

Individuals exposed to radiation or radiologically contaminated materials are at risk of developing life-threatening acute radiation syndrome (ARS), also known as radiation sickness. The clinical manifestations of ARS are dependent on radiation exposure levels, with damage to the bone marrow occurring at >2 Gy, gastrointestinal (GI) tract at >6 Gy, and neurovascular system at >10 Gy [1]. Bone marrow injury is coupled with neutropenia, leukopenia, and thrombocytopenia and is collectively known as the “hematopoietic subsyndrome of ARS” (H-ARS). Individuals that survive H-ARS often develop the delayed effects of acute radiation exposure (DEARE), which can manifest several months after the initial exposure and typically involve lung injury such as pneumonitis and pulmonary fibrosis [2,3,4]. The GI tract is also especially radiosensitive, and radiation exposure can result in damage to the GI epithelium, resulting in nutrient malabsorption, gut inflammation, and increased gut permeability, leading to translocation of gut bacteria into the blood stream [5]. When coupled with H-ARS, radiation-induced GI damage can lead to potentially fatal sepsis. Following exposure to high radiation doses, neurovascular damage can lead to blood–brain barrier damage, cerebral edema, and hemorrhaging.

There are currently six medical countermeasures (MCMs) approved by the United States Food and Drug Administration (US FDA) that can treat radiation injury. These MCMs include the leukocyte growth factors filgrastim (Neupogen), filgrastim biosimilar (Nypozi), pegfilgrastim (Neulasta), pegfilgrastim biosimilars (Udenyca, Stimufend, Ziextenzo), and sargrasmostim (Leukine); and the thrombopoietin analog romiplostim (Nplate) [6,7,8,9,10,11,12,13,14,15,16,17]. Unfortunately, these MCMs are only indicated to treat H-ARS, specifically radiation-induced neutropenia and/or thrombocytopenia, and they are only effective when given shortly after radiation exposure. During a radiological/nuclear event, first responders and military personnel may become exposed to high doses of radiation, and it is critical that they are protected from the harmful effects of radiation exposure. No radioprotector has yet been approved by the US FDA, although there are several under development [18,19]. BIO 300 Oral Powder (BIO 300) is an MCM that is currently under advanced development as an agent that can prophylactically protect individuals from the deleterious effects of radiation exposure [20]. The active ingredient in BIO 300 is synthetic genistein (5,7-dihydroxy-3-(4-hydroxyphenyl)-chromen-4-one), which functions as a non-steroidal, selective agonist of estrogen receptor beta (ERβ) [21]. BIO 300 has been shown to significantly improve survival in animals and prevent radiation-induced lung tissue damage when dosed orally for 2–6 days prior to lethal TBI [20,22]. Development of BIO 300 is regulated by the FDA Animal Rule, which requires drug efficacy to be established in one or two animal species, safety studies to be conducted in healthy humans, and an ability to bridge the animal effective dose to humans [23]. The Animal Rule lays out several methods for selection of an effective human dose, one of which is a biomarker-based approach. To utilize this approach, a biomarker(s) needs to be identified that is related to the drug’s mechanism of action, and there needs to be the ability to assess drug doses in humans that result in biomarker levels that are also associated with efficacy in animal studies. Recently, we have published transcriptomic changes in response to total- and partial-body gamma irradiation in a rhesus macaque model using lung and jejunum tissues [24,25].

Several studies have been completed in mice and nonhuman primates (NHPs) to assess metabolomic/lipidomic and proteomic biomarkers of BIO 300 [26,27]. Additionally, a phase 1 human safety study of BIO 300 evaluated transcriptomic changes in the whole blood of individuals dosed orally with BIO 300 [28]. Metabolomic and lipidomic analyses of blood samples have shown transient BIO 300-related changes in amino acid metabolites involved in cell health and immunity and metabolites involved in anti-oxidant activity and fatty acid metabolism [26]. However, the magnitude of these changes is often less than 10%, and high intra-group variance makes metabolomic biomarkers difficult to use for human dose selection. Proteomic studies in NHPs have shown BIO 300-related changes in proteins involved in oxidative stress response, innate immunity, and pro-inflammatory cytokine regulation [27]. Furthermore, transcriptomic analyses of whole blood from humans dosed with BIO 300 for six days showed significant changes in genes involved in inflammation, fatty acid metabolism, senescence, and hematopoietic cell regulation [28]. Given that BIO 300’s target, ERβ, acts as a transcription factor, analyses of blood-based gene and protein expression may be promising avenues for identification of a biomarker(s) for human dose selection.

In the present study, transcriptomic changes in the whole blood of CD2F1 mice treated with BIO 300 for six days were evaluated in the presence and absence of lethal TBI. Mice were dosed with three different doses of drug, including a known efficacious dose. Whole blood was collected for RNA sequencing 24–48 h after the last dose of BIO 300 in the absence of irradiation or 24 h after TBI. BIO 300 dose-dependent and TBI-dependent changes in differential gene expression and signaling pathways were profiled. The goal of this study was to identify signaling pathways that are related to BIO 300’s mechanism of action and determine putative biomarkers that can be used for human dose selection. Ideal biomarkers for human dose selection will be modulated by BIO 300 with and without irradiation because they will also need to be detectable in healthy humans.

## 2. Results

### 2.1. Profiling Whole-Blood Differential Gene Expression and Changes in Signaling Pathways in BIO 300-Treated Mice

**Figure 1:**
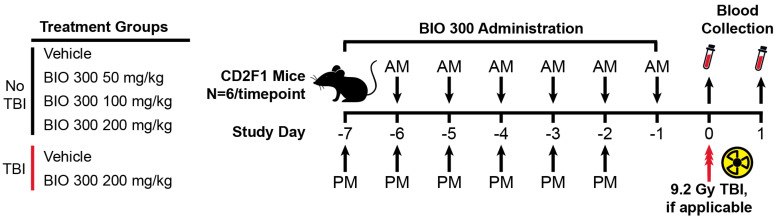
Study design. Male CD2F1 mice were assigned to one of six treatment groups. Animals were dosed with the indicated dose of BIO 300 or vehicle for six days, BID, for a total of 12 doses. Each dose was 12 h apart [morning dose (AM) and evening dose (PM)]. Animals were euthanized (N = 6/timepoint) 24 h and 48 h after their last dose of BIO 300 or vehicle, and whole blood was collected for transcriptomic analyses. Animals in the TBI groups were exposed to 9.2 Gy 24 h after their last dose of BIO 300 or vehicle. For animals with TBI, N = 6 animals were euthanized 24 h after TBI and whole blood was collected for transcriptomic analyses.

**Figure 2:**
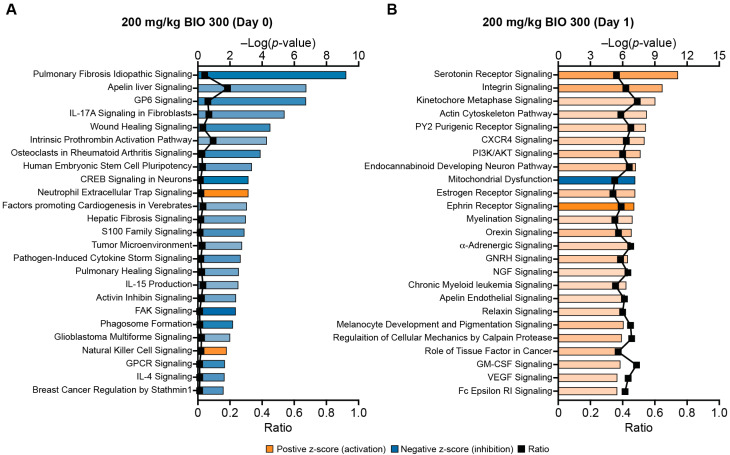
Top enriched signaling pathways in mice treated with a 200 mg/kg dose of BIO 300. Top 25 canonical pathways identified using IPA that have a Fisher’s exact test *p* < 0.05 and an absolute z-score > 2 in animals treated with BIO 300 (200 mg/kg, BID) at (**A**) 24 h (Day 0) and (**B**) 48 h (Day 1) after their last dose of BIO 300. Gene expression was compared to the respective vehicle groups in order to determine the top predicted activated or inhibited pathways. Blue bars indicate predicted inhibition of the pathway and orange bars indicate predicted activation. The darker the shading of the bars, the higher the absolute z-score. The black symbols represent the ratio of enriched genes in the data set compared to the total number of genes associated with the pathway.

Whole blood samples for RNA sequencing (RNAseq) were collected from mice on Day 0 and Day 1 (Figure 1). Comparisons between treatment groups for significant DEGs ranged from no observed DEGs to several thousand significant DEGs depending on the comparison groups (Table 1). BIO 300 dose-dependent effects on gene expression and signaling pathways were profiled. There were substantially fewer significant DEGs detected in animals that received 50 mg/kg/day of BIO 300 compared to those that received 100–200 mg/kg/day (Table 1). The majority of significant DEGs observed in the 50 mg/kg/day group were detected on Day 0, 24 h after the last dose of BIO 300, and few DEGs were identified on Day 1, 48 h after the last dose. This observation also extended to modulated signaling pathways on Day 0 and Day 1 for this dosing group (Supplementary Figure S1). The opposite pattern was observed for the 100 and 200 mg/kg/day groups where almost no significant DEGs were detected on Day 0, but over 7000 DEGs were reported on Day 1. Interestingly, the 100 and 200 mg/kg/day groups shared over 90% of the same significant DEGs on Day 1, and 17 of the top 25 activated/inhibited signaling pathways for each dosing group were the same (Figure 2, Supplementary Figure S2).

While only three genes met the significant DEG cutoff criteria (adjusted *p* < 0.05; absolute log2 fold change > 1), there were a number of signaling pathways that were significantly modulated (*p* < 0.05; absolute z-score > 2) in the 100 and 200 mg/kg/day groups on Day 0. The majority of the top 25 significantly modulated pathways in the 200 mg/kg/day group on Day 0 had predicted pathway inhibition (Figure 2A). In contrast, the majority of the top 25 significantly modulated pathways in this group on Day 1 were predicted to be activated (Figure 2B).

Of note, the only pathway that was oppositely regulated between these two data sets was tumor microenvironment signaling, which was predicted to be inhibited on Day 0 and was predicted to be activated on Day 1 (not shown in Figure 2B, as it was a top 100 pathway). Similar trends in pathways predicted to be inhibited versus activated were observed in the 100 mg/kg/day group (Supplementary Figure S2).

### 2.2. BIO 300 Attenuated Radiation-Induced Transcriptome Changes

**Figure 3:**
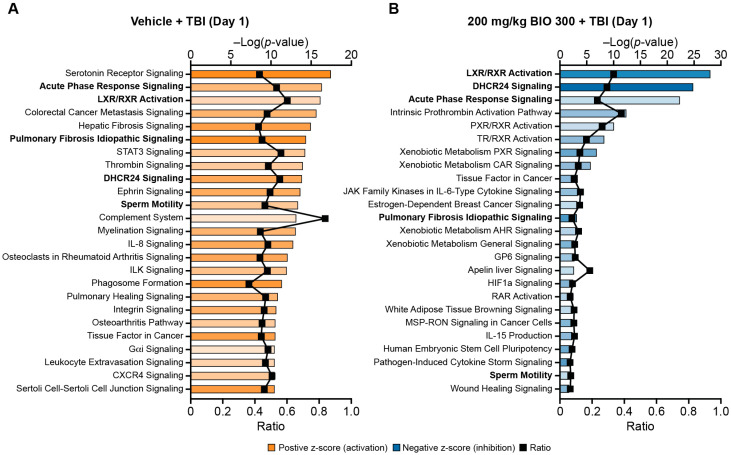
Top enriched signaling pathways in mice treated with vehicle or BIO 300 with TBI. Top 25 canonical pathways identified using IPA that have a Fisher’s exact test *p* < 0.05 and an absolute z-score > 2 in animals treated with vehicle or BIO 300 (200 mg/kg, BID) prior to TBI. Sampling occurred 24 h after TBI (48 h after the last dose of study treatment; Day 1). (**A**) Gene expression in vehicle-treated animals with TBI was compared to Day 1 of vehicle-treated animals that were not exposed to radiation. (**B**) Gene expression of BIO 300-treated animals exposed to radiation was compared to Day 1 of vehicle-treated animals with TBI. Blue bars indicate predicted inhibition of the pathway and orange bars indicate predicted activation. The darker the shading of the bars, the higher the absolute z-score. The black symbols represent the ratio of enriched genes in the data set compared to the total number of genes associated with the pathway. Inversely enriched pathways that appeared in the top 25 list in both the vehicle and BIO 300 group are bolded.

Profiling gene expression differences between vehicle and BIO 300-treated animals that were exposed to radiation revealed distinct differences in signaling pathway networks. In the comparison of the transcriptome profile of vehicle treated animals on Day 1, 24 h after 9.2 Gy TBI to the profile of nonirradiated, vehicle-treated animals on Day 0 (24 h after the last dose) or Day 1 (48 h after the last dose) showed significant activation of radiation-response-related signaling pathways in animals exposed to radiation, such as acute phase response signaling, pulmonary fibrosis idiopathic signaling, thrombin signaling, complement system, and interleukin 8 (IL-8) signaling, which were all present in the top 25 enriched pathways (Figure 3A).

Additional radiation-response-related pathways that were outside of the top 25 but still significantly activated (*p* < 0.05; z score > 2) in the vehicle-treated/radiation-exposed animals included pathogen-induced cytokine storm signaling, transforming growth factor-β (TGF-β) signaling, production of nitric oxide and reactive oxygen species in macrophages, and IL-1 signaling. The majority of signaling pathways significantly modulated in BIO 300 (200 mg/kg)-treated/TBI animals were inhibited, including those pathways that were activated in vehicle-treated animals such as acute phase response signaling, pulmonary fibrosis idiopathic signaling, and LXR/RXR activation (Figure 3B). No pathways were identified in the radiation-exposed vehicle and BIO 300 (200 mg/kg) groups that were significantly modulated in the same direction; *p* < 0.05 and absolute z score > 2 in both groups.

**Figure 4:**
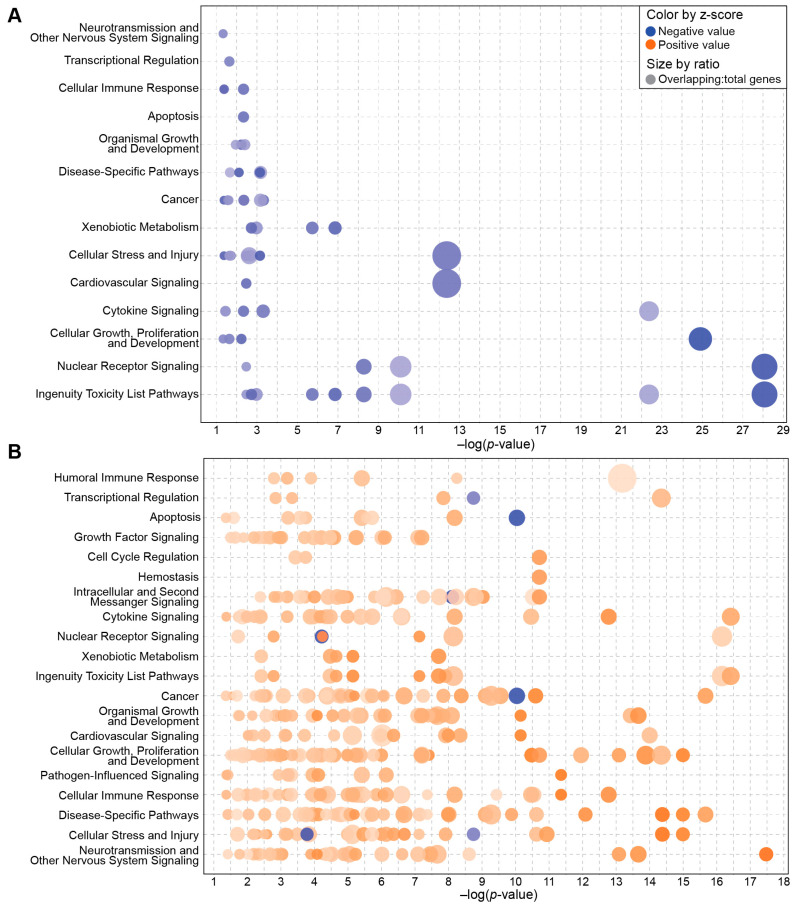
Pathway categories containing significantly enriched signaling pathways in vehicle or BIO 300 treated mice with TBI. Bubble chart depicting the signaling pathways within specific pathway categories. (**A**) Enriched pathways on Day 1 in BIO 300 (200 mg/kg, BID) treated mice with TBI compared to vehicle-treated animals with TBI. (**B**) Enriched pathways on Day 1 in vehicle-treated mice with TBI compared to unirradiated, vehicle-treated mice. Each bubble represents a different signaling pathway, blue bubbles are inhibited pathways and orange bubbles are activated pathways with darker shading indicating higher absolute z-scores. The size of each bubble is based on the ratio of enriched genes in the data set compared to the total number of genes associated with the pathway. The larger the bubble, the higher the ratio. The signaling pathways within each pathway category are provided in Supplementary Tables S1 and S2 for panel A and B, respectively.

In the comparison of the categories of pathways enriched in animals exposed to radiation after vehicle or BIO 300 treatment, it was evident that there was a higher abundance of significantly enriched (*p* < 0.05 and absolute z score > 2) pathways in vehicle-treated/radiation-exposed mice (Figure 4). Inhibited pathways in BIO 300-treated/radiation-exposed animals were associated with pathway categories including cellular immune response; cytokine signaling; cellular stress and injury; and cellular growth, proliferation, and development (Figure 4A, Supplementary Table S1). Conversely, in vehicle-treated/radiation-exposed animals, these pathway categories were also enriched, but with activated signaling pathways (Figure 4B, Supplementary Table S2).

### 2.3. Identifying Putative Biomarkers of BIO 300 for Human Dose Selection

**Figure 5:**
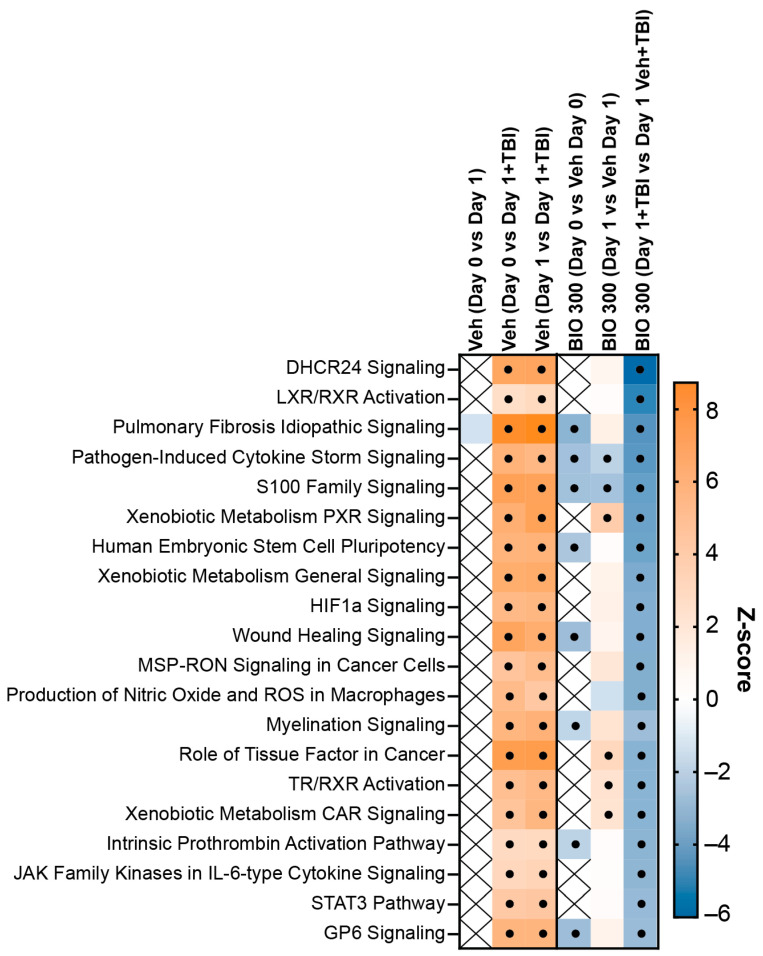
Comparison of enriched pathways in BIO 300 and vehicle-treated animals with or without TBI. The top 20 pathways by z-score with Fisher’s exact test *p* < 0.05 from mice treated with BIO 300 (200 mg/kg, BID) and with TBI were compared to Day 0 and Day 1 of unirradiated animals treated with BIO 300 (200 mg/kg, BID) and vehicle-treated animals that were unirradiated or exposed to radiation. Shaded squares indicated z-score (blue, negative z-score; orange, positive z-score). Squares with an ‘X’ indicate that the pathway was not enriched to any degree within the specified group. A black dot indicates that the absolute z-score value is >2.

Ideal biomarkers for human dose selection are modulated in the same direction by BIO 300 in both normal and radiation-exposed animals. To identify putative biomarkers specifically modulated by BIO 300 in the presence and absence of TBI, significantly regulated signaling pathways were compared between BIO 300 (200 mg/kg)-treated animals on Day 0 and Day 1 and BIO 300 (200 mg/kg)-treated animals exposed to radiation on Day 1. Signaling pathways were also compared between Day 0 and Day 1 of vehicle treated animals with and without TBI with the assumption that signaling pathways affected by radiation should be modulated in the same direction (activation or inhibition) on Day 0 and Day 1. Results indicated that only two pathways were significantly (absolute z-score ≥ 2) activated in vehicle-treated/radiation-exposed animals and significantly inhibited in BIO 300 (200 mg/kg)-treated animals with and without TBI (Figure 5).

These pathways were two inflammatory signaling pathways: pathogen-induced cytokine storm signaling and S100 family signaling. Both of these pathways were unchanged from Day 0 to Day 1 in the vehicle-treated animals that were not exposed to radiation. Predicted activation of S100 pathway signaling in irradiated, vehicle-treated animals and predicted inhibition with irradiated, BIO 300-treated animals was likely driven by a log2 fold change in gene expression > 2 for the *S100B* gene in both vehicle comparison groups and by a −2 log2 fold change in irradiated, BIO 300-treated animals (Supplementary Table S3). Downregulation of S100 proteins likely had radioprotective effects, as the S100 proteins are mediators of inflammatory response due to various stimuli and S100B has been previously shown to be upregulated in response to irradiation [29,30]. Similar results were reported for *EGFR* and *FGFR* family members 1–4, two cell-surface receptors that are activated by *S100B*. For the cytokine storm signaling pathway, toll-like receptor genes (*TLR2*, *TLR3*, *TLR4*, *TLR5*, *TLR6*, and *TLR8*) had a >1 log2 fold change in irradiated, vehicle-treated animals, while the log2 fold change in BIO 300-treated animals was below 0 for the 6x *TLR* genes (Supplementary Table S4). Toll-like receptors are induced by irradiation, so repression of these genes by BIO 300 may be a direct mechanism for mitigation of radiation-induced inflammation [31]. Additional differentially regulated genes in this pathway that were upregulated in irradiated, vehicle-treated animals and downregulated or unchanged in BIO 300-treated animals included completement system genes *C3* and *C5*, collagen gene family members, fibrinogen family members, and various cytokines. Downregulation of these inflammatory genes further reinforce the anti-inflammatory effects of BIO 300 and suggest a mechanism for radioprotection.

### 2.4. Comparing Transcriptome Changes between BIO 300-Treated Mice and Humans

**Figure 6:**
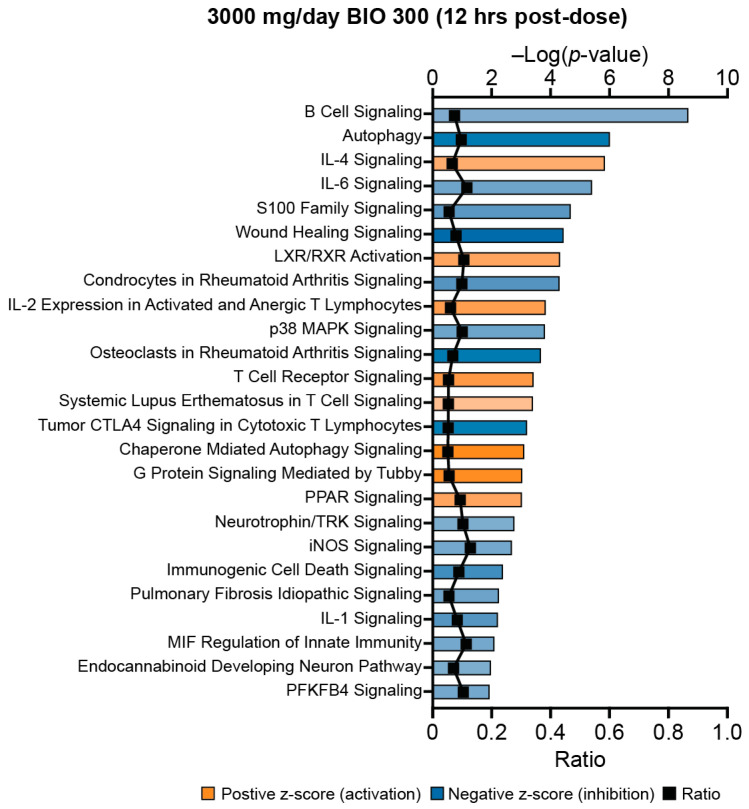
Top enriched signaling pathways in healthy volunteers treated with BIO 300 Oral Powder. Top 25 canonical pathways identified using IPA that have a Fisher’s exact test *p* < 0.05 and an absolute z-score > 2 in healthy volunteers treated with BIO 300 once daily at 3000 mg/day for six consecutive days. Whole blood was sampled at 12 h after the last dose for transcriptomic analyses and changes in gene expression were compared to whole blood sampled just prior to the first dose. Data were adapted from a previously published phase 1 clinical trial of healthy volunteers. Blue bars indicate predicted inhibition of the pathway and orange bars indicate predicted activation. The darker the shading of the bars, the higher the absolute z-score. The black symbols represent the ratio of enriched genes in the data set compared to the total number of genes associated with the pathway.

RNA sequencing data were analyzed from a completed phase 1 clinical study of BIO 300 in healthy volunteers [28]. The top 25 significantly inhibited or activated pathways at 12 h after six days of once-daily dosing included several pro-inflammatory pathways that were predicted to be significantly inhibited (*p* < 0.05; z-score < −2) (Figure 6). There were several overlapping pathways with the mouse dataset from irradiated, BIO 300-related animals, including S100 signaling, wound-healing signaling, and pulmonary fibrosis idiopathic signaling. Interestingly, while inhibition of S100 signaling in mice appears to be driven by the downregulation of *S100B*, in humans, it is driven by downregulation of *S100A8*/*S100A9*. In both the wound-healing signaling and pulmonary fibrosis idiopathic signaling pathways, *TGF-α* was significantly downregulated in both the mouse and human datasets.

## 3. Discussion

Development of novel radiation medical countermeasures that can prophylactically protect individuals from the deleterious effects of radiation exposure requires a practical and feasible approach to translating the effective dose in animals to humans. One such approach outlined in the FDA Animal Rule is a “biomarker approach” where a biomarker related to the agent’s mechanism of action is modulated to a desired level in humans based on the observed biomarker level correlating with a therapeutic effect in animals [32]. Here, we propose to assess molecular changes in the whole blood by RNA sequencing since both radiation exposure and activation of estrogen receptor beta, a transcription factor and the molecular target for genistein, can cause a cascade of gene expression changes.

The main objective of the study was to determine dose-dependent changes in gene expression caused by BIO 300 and to determine the differential effect(s) of BIO 300 on gene expression in animals exposed to radiation. Due to blood collection requiring terminal bleeds, survival was not a component of the study; however, dose levels of BIO 300 were chosen based on previous efficacy studies conducted in CD2F1 mice. A 200 mg/kg BID dose is an efficacious dose capable of significantly improving survival in mice when given prior to TBI. Therefore, we anticipated that a 200 mg/kg BID dose would cause significant and relevant changes in gene expression [20]. A 50 mg/kg BID dose has previously been shown to be ineffective, and therefore, 100 mg/kg was viewed as an appropriate intermediate dose. These three dose levels allowed for the potential detection of dose-dependent changes in gene expression. Animals were dosed for 6 consecutive days because this was determined to be the optimal dose schedule for radioprotection by oral formulation of BIO 300 [20]. Animals dosed with 100 or 200 mg/kg BIO 300 without TBI had thousands of significantly differential expressed genes (DEGs) detected on Day 1 (48 h after the last dose of drug) compared to vehicle-treated animals, whereas the 50 mg/kg group had the majority of DEGs detected on Day 0 (24 h after the last dose of drug), which was still considerably fewer DEGs than that of the other dosing groups. These findings support the conclusion that 50 mg/kg is an ineffective dose in mice and suggest that 100 mg/kg might be similarly effective as a 200 mg/kg dose. It is surprising that almost no DEGs were identified in the 100 and 200 mg/kg dosing groups on Day 0, since this timepoint corresponds with the time of irradiation in efficacy studies and the 200 mg/kg dose has previously shown to be efficacious in this model. Considering that DEGs were identified in humans at 12 h after six daily doses, it is possible that significant transcriptomic changes occurred earlier than Day 0, and future studies should investigate additional timepoints between Day 1 and Day 0. In irradiated animals, both vehicle- and 200 mg/kg BIO 300-treated animals had thousands of significant DEGs on Day 1 post-TBI. Interestingly irradiation caused more upregulated genes, and the BIO 300 treatment alone resulted in more downregulated genes. Given the substantial quantity of DEGs identified across the different treatment groups, we used pathway analyses to determine which signaling pathways were being affected by drug treatment and radiation exposure.

Assessment of the top 25 signaling pathways in unirradiated animals treated with 100 or 200 mg/kg BIO 300 revealed that a host of inflammatory and wound-healing pathways were predicted to be inhibited on Day 0 (24 h after the last dose). Conversely, on Day 1 (48 h after the last dose), the majority of the top 25 regulated pathways were predicted to be activated and include pathways involved in hematopoiesis, immunity, and platelet function, and pathways involved in cell growth and survival. This is consistent with previously published studies that have demonstrated that anti-inflammatory effects of genistein [33,34,35]. Additionally, in a phase 1 clinical trial of BIO 300 in healthy male and female volunteers, a 3000 mg/day dose also was predicted to significantly inhibit several pro-inflammatory pathways such as S100 signaling and wound healing signaling [28]. A 3000 mg dose in humans is equivalent to approximately 600 mg/kg in mice based on body surface area [36]. Importantly, while these inhibitory signaling changes appear to be related to the drug’s mechanism of action, there is no current evidence that suggest these changes have adverse side effects in unirradiated individuals. Studies of oral formulations of BIO 300 in healthy volunteers and patients with NSCLC have only reported mild-to-minor gastrointestinal adverse events following up to 8 weeks of daily dosing, which is not unexpected for an oral drug [28,37].

Comparison of the top 25 signaling pathways significantly enriched in vehicle or BIO 300 (200 mg/kg)-treated animals 24 h after TBI indicated that BIO 300 inhibits pathways that are activated by radiation. From the evaluation of the categories of pathways enriched in animals with TBI and treated with vehicle or BIO 300, it is evident that the vehicle animals have considerably more enriched pathways, which may suggest that BIO 300 helps animals better maintain homeostasis. Although there were no significant DEGs per our criteria (adjusted *p* < 0.05; absolute log2 fold change > 1) between irradiated vehicle-treated animals and irradiated BIO 300-treated animals, animals treated with BIO 300 had enriched signaling pathways moving in the opposite direction compared to vehicle-treated animals. In order to identify pathways specific to BIO 300 and relevant to its radioprotective mechanism, we compared pathways enriched in vehicle-treated mice exposed to radiation and BIO 300-treated mice with and without TBI. Pathways that were modulated inversely to vehicle-treated animals exposed to radiation and observed in BIO-300 treated animals with or without TBI are ideal for identification of biomarkers for human dose selection. Since no individual significant DEGs were identified between irradiated vehicle-treated animals and irradiated BIO 300-treated animals, these pathways should be explored in future studies for potential biomarkers. Of the enriched pathways, only two pathways were significantly activated in vehicle/TBI animals and significantly inhibited in the BIO 300 ± TBI groups: pathogen-induced cytokine storm signaling and S100 family signaling. Additionally, two other pathways, pulmonary fibrosis idiopathic signaling and wound-healing signaling, were also similarly inhibited in the human transcriptomic dataset and in the BIO 300-treated/radiation-exposed animals, and on Day 0, in the BIO 300-treated alone animals.

Pathogen-induced cytokine storm signaling can lead to uncontrolled, systemic inflammation precipitated by excessive cytokine release from cells. Immune cells have pattern recognition receptors (PRRs) such as TLRs and C-type lectin receptors that recognize pathogen-associated molecular patterns (PAMPs) or damage-associated molecular patterns (DAMPs). DAMPs in particular are released by dead cells, such as those killed by radiation exposure, and stimulate immune cells to release pro-inflammatory cytokines. Of note, in the phase 1 clinical study of BIO 300, one of the top differentially expressed genes was *CLEC4E*, a C-type lectin receptor that functions as a DAMP and has been previously shown to be involved in radiation-induced cytokine signaling [38]. *CLEC4E* was significantly upregulated in vehicle-treated, irradiated mice (log2 fold change 2.26; adjusted *p* < 0.01) but was not differentially expressed in BIO 300-treated irradiated or unirradiated mice at any dose level in the present study.

Pulmonary fibrosis idiopathic signaling and wound healing signaling are pro-inflammatory/pro-fibrotic responses that involve the recruitment of immune cells and remodeling of the pulmonary extra cellular matrix. Activation of pulmonary fibrosis idiopathic signaling in damaged epithelial cells and immune cells results in the secretion of pro-inflammatory cytokines and tissue repair mediators, such as TGF-β. RNA sequencing of the GI tract of NHPs following TBI also showed pulmonary fibrosis idiopathic signaling to be significantly activated in irradiated animals [24]. Interestingly, a recent clinical study of a related genistein-based oral drug formulation, BIO 300 Oral Suspension, in patients with non-small cell lung cancer undergoing chemoradiotherapy demonstrated that patients had a BIO 300 Oral Suspension dose-dependent decrease in serum TGF-β levels [37]. BIO 300 Oral Suspension and BIO 300 have also been previously shown to prevent or mitigate radiation-induced lung injury in mice [22,39]. Pulmonary fibrosis idiopathic signaling and wound-healing signaling were also inhibited in a clinical study of BIO 300 in healthy volunteers [28]. These data indicate that BIO 300 is able to ablate the pro-inflammatory and/or pro-fibrotic processes induced by radiation exposure.

S100 signaling is driven by calcium-binding S100 proteins that function as DAMPs which activate immune function. S100 proteins have been previously shown to be upregulated by TBI in transcriptomic and proteomic datasets in NHPs and mice [24,40]. A previous proteomic study of a parenteral formulation of BIO 300 (BIO 300 Injectable Suspension) found that a single intramuscular injection resulted in a detectable downregulation of S100 in the serum of NHP [27]. Interestingly, in the present study, inhibition of S100 signaling was primarily driven by downregulation of *S100B* expression, while in a clinical study of BIO 300, it was driven by downregulation of *S100A8* expression [28]. Considering S100 proteins are secreted into the blood, allowing for the straightforward quantification by conventional methods such as protein ELISA, mediators of this pathway should be investigated as potential biomarkers of BIO 300.

A number of limitations are noted for this study. A major limitation is the use of single sex animals. This study was conducted with only male animals to reduce variability in transcriptomics data since there are known differences in radiosensitivity between male and female animals [41]. It will be critical to evaluate biomarkers in female animals in future studies to confirm findings. In addition, whole-blood sampling only occurred at two timepoints in unirradiated animals and only a single timepoint in irradiated animals, and these snapshots of differential gene expression may not be fully representative of all the effects caused by TBI and/or BIO 300 treatment. The study also utilized terminal bleeds for whole-blood sampling, and animals had to be anesthetized prior to blood collection, which might have impacted the transcriptome profile. Canonical pathway analysis for the mouse samples used a cutoff of absolute log2 fold change in gene expression > 1 regardless of *p*-value. This was done in order to ensure an adequate number of genes for analyses but could have led to noise in the datasets. Future studies are required to validate the findings presented here. Finally, validation of the results, such as by RT-qPCR or immunoblot, was not completed in the present study, and validation of key results will need to be considered for future studies.

In summary, we found that animals exposed to 9.2 Gy TBI had numerous pathways activated related to immune response and cellular growth and development, whereas animals treated with BIO 300 prior to TBI had fewer pathways enriched, and the signaling pathways involved in immune response, cellular stress, and cellular growth and development were significantly inhibited. Four pathways were activated in irradiated, vehicle-treated animals but inhibited in BIO 300-treated animals with or without TBI and inhibited in healthy humans treated with BIO 300: pathogen-induced cytokine storm signaling, pulmonary fibrosis idiopathic signaling, wound-healing signaling, and S100 family signaling. These pathways have potential links to BIO 300’s radioprotective mechanism of action, and mediators within these pathways should be explored as potential biomarkers for human dose selection.

## 4. Materials and Methods

### 4.1. Study Design

In order to interrogate transcriptome-based biomarkers of BIO 300, a two-part study was conducted in mice to collect whole-blood samples for RNA sequencing (RNAseq). The first part evaluated transcriptome changes following six days of dosing of three different dose levels in the absence of TBI, while the second part evaluated the known effective dose following six days of dosing prior to 9.2 Gy TBI. For this study, Day 0 corresponded to the time of TBI. As with previous nonclinical efficacy studies of BIO 300, TBI occurred 24 h after the last dose of drug 25–27. Thus, the Day 1 timepoint corresponded to 24 h after TBI and 48 h after the last dose of BIO 300. Importantly, a previous study of parenteral administration of BIO 300 showed that the drug is only effective 48 h after dosing 24. The 9.2 Gy TBI dose was selected because previous studies using CD2F1 mice demonstrated that a TBI dose of 9.2 Gy equates to a LD70/30 in this strain of mice [20].

### 4.2. Animal

Male CD2F1 (5–6-week-old) mice were purchased through Envigo (Indianapolis, IN, USA) and were housed (3 mice per cage) in an Association for Assessment and Accreditation of Laboratory Animal Care (AAALAC) International-accredited facility. The rooms that the mice were kept in had a 12 h light/dark cycle and were maintained at 21 ± 2 °C with 10–15 hourly cycles of fresh air and a relative humidity of 50 ± 10%. Mice were quarantined for one week following arrival and allowed to acclimate to the new housing, and representative fecal samples underwent microbiological examination to ensure that the animals were free of *Pseudomonas aeruginosa*. Certified rodent rations (Teklad Rodent Diet, Harlan Laboratories, Inc., Indianapolis, IN, USA) and acidified water (HCl, pH = 2.5–2.8) were provided ad libitum. All procedures were performed following a protocol approved by the Armed Forces Radiobiology Research Institute’s (AFRRI, Bethesda, MA, USA) Institutional Animal Care and Use Committee (IACUC; Protocol # PHA-20-026 approved on 10 June 2020). All experiments were performed in accordance with relevant guidelines and regulations. This study is reported in accordance with the ARRIVE guidelines.

### 4.3. Total-Body Irradiation

The high-level cobalt facility (HLCF) used for TBI has the exposure room to provide shielding for up to 400,000 curies. The HLCF is categorized as a wet-storage panoramic dry irradiator (radiation sources stored underwater and raised for irradiation). The mice were placed in compartmentalized Plexiglas irradiation boxes, designed to hold eight mice per box. Only twelve mice were irradiated, with six placed in each box, along with phantoms. The boxes were secured in irradiation towers before being transferred to the irradiation platform. The mice were exposed to a bilateral, midline dose of 9.2 Gy total-body ^60^Co γ-radiation at a dose rate of 0.6 Gy/min. The radiation dosimetry used in this study has been discussed in previous publications [20]. Following irradiation, mice were observed for any adverse reactions before being returned to their home cages.

### 4.4. Drug Preparation and Dosing

BIO 300 Oral Powder (BIO 300) is an amorphous solid dispersion of synthetic genistein produced by hot melt extrusion resulting in a free-flowing powder (provided by Humanetics Corporation, Minneapolis, MN, USA). The powder contains 35% genistein and 65% Kollidon 12 PF (*w*/*w*). A mortar and pestle was used to mix BIO 300 with its vehicle to deliver 0.2 mL of either a 50, 100, or 200 mg/kg dose (herein, referred to with mg genistein/kg animal weight). The vehicle was composed of water, methylcellulose (0.5% *w*/*v*), and polyvinylpyrrolidone K25 (3% *w*/*v*). The solution, after preparation, was stirred using a magnetic stirrer in a beaker at room temperature continuously for at least 2 h prior to dosing. A 1 mL Luer-lock syringe with a 20 G disposable feeding needle with a silicone-rubber tip was used to deliver the drug or vehicle administrations via po route. For po drug administration, the mice were secured by their scruff in order to extend the two front legs and immobilize the head and neck. The gavage needle was carefully inserted into the side of the subject’s mouth and guided along the hard palate of the mouth to the back of the throat until it reached the abdominal area to deliver the bolus. Between dosing, 70% ethanol (absolute alcohol diluted to 70%) was used to disinfect the feeding needle, and the needle was then wiped with purified water to prevent irritation to the mucosa and cross contamination to other animals from the same housing cage.

### 4.5. Blood Sample Collection

Blood was collected from six mice per timepoint. Briefly, whole blood (1 mL) was collected from each anesthetized (Isoflurane, Abbott Laboratories, Chicago, IL, USA) mouse via the inferior vena cava using a 23-gauge needle into a PAXgene blood RNA tube (PreAnalytiX, a Qiagen/Becton, Dickinson and Company, Franklin Lakes, NJ, USA). The blood was mixed immediately by inverting the tube 10 times. The tubes were left at room temperature on the bench overnight and subsequently stored at −80 °C until used. Following blood collection, the mice were euthanized.

### 4.6. RNA Extraction

Total RNA was isolated from each whole blood sample following the manufacturer’s protocol for the PAXgene Blood RNA Kit (PreAnalytiX, Hombrechtikon, Switzerland) and quantified by fluorometry using Qubit 4 fluorometer (Invitrogen, Carlsbad, CA, USA). The quality of RNA was analyzed on a Bioanalyzer Eukaryote Total RNA Pico Chip (Agilent 2100, Agilent, CA, USA) [42]. The average RNA integrity number (RIN) score across all samples was above the recommended minimum RIN of 7. Total RNA samples were stored at −80 °C until used. All RNA samples extracted from whole blood had an RNA integrity number (RIN) ≥ 6.1, and 97% of samples had an RIN ≥ 7.1, indicating good quality with minimal degradation. This was especially important for the samples collected from irradiated animals, which had an RIN ≥ 7.1 (only a single sample was below 7 (6.9)). RNAseq yielded over 29 million reads in every sample, and 95% of the samples produced over 41 million reads per sample.

### 4.7. RNA Sequencing and Analyses

The library for RNA-Seq was prepared with 600 ng of total RNA input using TrueSeq stranded Total RNA with Ribo-Zero Globin kit (Illumina, San Diego, CA, USA) with barcoded adapters. There were 6 biological replicates per timepoint. Library size distribution was determined using a Bioanalyzer DNA 1000 kit (Agilent 2100, Agilent, CA, USA), and the library yield and concentration were determined using the KAPA Library Quantification Kit for Illumina (Kapa Biosystems, Inc. Wilmington, MA, USA). Sequencing reads were evaluated for quality, and poor-quality reads were trimmed using the next-generation sequencing processing software, Trimmomatic v.0.36 [43]. Following trimming, the reads were mapped to the *Mus musculus* GRCm38 reference genome using STAR aligner v.2.5.2b [44]. Over 20 million unique mapped reads were generated per sample. Unique gene hit counts were calculated using the Subread package v.1.5.2, and differential expression analysis was performed using DESeq2 [45,46]. A Wald test was used to determine *p*-values for log2 fold change in expression for pre-specified comparisons. In order to control for the false discovery rate (FDR of 0.1), the *p*-value was adjusted using the Bonferroni–Hochberg method to control for multiple comparisons. Genes with an adjusted *p*-value < 0.05 and absolute log2 fold change > 1 were considered significantly differentially expressed genes (DEGs).

### 4.8. Signaling Pathway Analyses

RNA sequencing data were analyzed for canonical pathway analyses with use of Qiagen Ingenuity Pathway Analysis (IPA) (QIAGEN Inc., Germantown, MD, USA, https://digitalinsights.qiagen.com/IPA, accessed on 10 July 2024) [47]. Canonical pathways analysis identified pathways using the QIAGEN IPA library of canonical pathways that were enriched in the data set. Genes from the mouse data set that met the absolute log2 fold change cutoff of 1 and were associated with a canonical pathway in the QIAGEN Knowledge Base were considered for the analysis. For analysis of the previously published human dataset, a cutoff of absolute log2 fold change > 0.5 was used to ensure 100–3000 genes were in the dataset for pathway analysis. The significance of the association between the data set and the canonical pathway was measured in three ways: (1) a ratio of the number of molecules from the data set that map to the pathway was divided by the total number of molecules that map to the canonical pathway is displayed; (2) a right-tailed Fisher’s Exact Test was used to calculate a *p*-value for determining the probability that the association between the genes in the dataset and the canonical pathway is explained by chance alone; and (3) a z-score was calculated to indicate the likelihood of inhibition or activation of that pathway, with z-scores greater than −2 or 2 indicating significant inhibition or activation of the pathway, respectively.

## Figures and Tables

**Table 1:** Significantly differentially expressed genes.

Control	Treatment	Up	Down	Total DEGs
Vehicle–Day 1	BIO 300 (200 mg/kg)–Day 1	3555	4125	7680
Vehicle–Day 1	BIO 300 (100 mg/kg)–Day 1	3612	4036	7648
Vehicle–Day 1	Vehicle + TBI–Day 1	4506	2676	7182
Vehicle–Day 0	Vehicle + TBI–Day 1	3244	2146	5390
Vehicle–Day 0	BIO 300 (50 mg/kg)–Day 0	8	1131	1139
Vehicle–Day 1	BIO 300 (50 mg/kg)–Day 1	35	20	55
Vehicle–Day 0	Vehicle–Day 1	1	37	38
Vehicle–Day 0	BIO 300 (200 mg/kg)–Day 0	3	0	3
Vehicle–Day 0	BIO 300 (100 mg/kg)–Day 0	0	2	2
Vehicle + TBI–Day 1	BIO 300 (200 mg/kg) + TBI–Day 1	0	0	0

Significantly Differentially expressed genes from RNA-seq data using *DESeq2* analysis are shown. Significance based on adjusted *p* < 0.05 and absolute log2 fold change > 1. *DEGs*, differentially expressed genes; *TBI*, total-body irradiation.

## Data Availability

The data discussed in this publication have been deposited in NCBI’s Gene Expression Omnibus and are accessible through GEO Series accession number GSE268247. The data can be directly accessed via the link (https://www.ncbi.nlm.nih.gov/geo/query/acc.cgi?acc=GSE268247, accessed on 10 July 2024).

## Supplementary Materials

The following supporting information can be downloaded at https://www.mdpi.com/article/10.3390/ijms25168818/s1.

## References

[1] Singh V.K., Seed T.M. (2017). A review of radiation countermeasures focusing on injury-specific medicinals and regulatory approval status: Part I. Radiation sub-syndromes, animal models and FDA-approved countermeasures. Int. J. Radiat. Biol..

[2] Van Dyk J., Keane T., Kan S., Rider W., Fryer C. (1981). Radiation pneumonitis following large single dose irradiation: A re-evaluation based on absolute dose to lung. Int. J. Radiat. Oncol. Biol. Phys..

[3] Gross N.J. (1977). Pulmonary Effects of Radiation Therapy. Ann. Intern. Med..

[4] Williams J.P., Jackson I.L., Shah J.R., Czarniecki C.W., Maidment B.W., DiCarlo A.L. (2012). Animal models and medical countermeasures development for radiation-induced lung damage: Report from an NIAID workshop. Radiat. Res..

[5] Elliott T.B., Deutz N.E., Gulani J., Koch A., Olsen C.H., Christensen C., Chappell M., Whitnall M.H., Moroni M. (2014). Gastrointestinal acute radiation syndrome in Göttingen minipigs (*Sus scrofa domestica*). Comp. Med..

[6] Farese A., MacVittie T. (2015). Filgrastim for the treatment of hematopoietic acute radiation syndrome. Drugs Today.

[7] Singh V., Seed T. (2018). An update on sargramostim for treatment of acute radiation syndrome. Drugs Today.

[8] Hankey K.G., Farese A.M., Blaauw E.C., Gibbs A.M., Smith C.P., Katz B.P., Tong Y., Prado K.L., MacVittie T.J. (2015). Pegfilgrastim Improves Survival of Lethally Irradiated Nonhuman Primates. Radiat. Res..

[9] Clayton N.P., Khan-Malek R.C., Dangler C.A., Zhang D., Ascah A., Gains M., Gardner B., Mockbee C., Keutzer J.M., McManus J. (2021). Sargramostim (rhu GM-CSF) Improves Survival of Non-Human Primates with Severe Bone Marrow Suppression after Acute, High-Dose, Whole-Body Irradiation. Radiat. Res..

[10] Zhong Y., Pouliot M., Downey A.-M., Mockbee C., Roychowdhury D., Wierzbicki W., Authier S. (2021). Efficacy of delayed administration of sargramostim up to 120 hours post exposure in a nonhuman primate total body radiation model. Int. J. Radiat. Biol..

[11] Farese A.M., Cohen M.V., Katz B.P., Smith C.P., Gibbs A., Cohen D.M., MacVittie T.J. (2013). Filgrastim Improves Survival in Lethally Irradiated Nonhuman Primates. Radiat. Res..

[12] Lazarus H.M., McManus J., Gale R.P. (2022). Sargramostim in acute radiation syndrome. Expert Opin. Biol. Ther..

[13] Wong K., Chang P.Y., Fielden M., Downey A.M., Bunin D., Bakke J., Gahagen J., Iyer L., Doshi S., Wierzbicki W. (2020). Pharmacodynamics of romiplostim alone and in combination with pegfilgrastim on acute radiation-induced thrombocytopenia and neutropenia in non-human primates. Int. J. Radiat. Biol..

[14] Bunin D.I., Javitz H.S., Gahagen J., Bakke J., Lane J.H., Andrews D.A., Chang P.Y. (2023). Survival and Hematologic Benefits of Romiplostim after Acute Radiation Exposure Supported FDA Approval under the Animal Rule. Int. J. Radiat. Oncol. Biol. Phys..

[15] U.S. Food and Drug Administration (2024). Radiological and Nuclear Emergency Preparedness Information from FDA. https://www.fda.gov/emergency-preparedness-and-response/mcm-issues/radiological-and-nuclear-emergency-preparedness-information-fda.

[16] Fresenius Kabi (2023). STIMUFEND (Pegfilgrastim-Fpgk) is Biosimilar* to NEULASTA (pegfilgrastim). https://www.fda.gov/media/172679/download?attachment.

[17] Coherus BioSciences Inc (2022). UDENYCA (Pegfilgrastim-Cbqv) is Biosimilar* to NEULASTA (Pegfilgrastim). https://www.accessdata.fda.gov/drugsatfda_docs/label/2022/761039s014lbl.pdf.

[18] Singh V.K., Seed T.M. (2023). Development of gamma-tocotrienol as a radiation medical countermeasure for the acute radiation syndrome: Current status and future perspectives. Expert Opin. Investig. Drugs.

[19] Kiang J.G., Cannon G., Singh V.K. (2024). An Overview of Radiation Countermeasure Development in Radiation Research from 1954 to 2024. Radiat. Res..

[20] Singh V.K., Fatanmi O.O., Wise S.Y., Carpenter A., Nakamura-Peek S., Serebrenik A.A., Kaytor M.D. (2022). A novel oral formulation of BIO 300 confers prophylactic radioprotection from acute radiation syndrome in mice. Int. J. Radiat. Biol..

[21] Landauer M.R., Harvey A.J., Kaytor M.D., Day R.M. (2019). Mechanism and therapeutic window of a genistein nanosuspension to protect against hematopoietic-acute radiation syndrome. J. Radiat. Res..

[22] Singh V.K., Serebrenik A.A., Fatanmi O.O., Wise S.Y., Carpenter A.D., Janocha B.L., Kaytor M.D. (2023). The Radioprotectant, BIO 300, Protects the Lungs from Total-Body Irradiation Injury in C57L/J Mice. Radiat. Res..

[23] U.S. Food and Drug Administration (2015). Guidance Document: Product Development under the Animal Rule. http://www.fda.gov/downloads/drugs/guidancecomplianceregulatoryinformation/guidances/ucm399217.pdf.

[24] Vellichirammal N.N., Sethi S., Avuthu N., Wise S.Y., Carpenter A.D., Fatanmi O.O., Guda C., Singh V.K. (2023). Transcriptome profile changes in the jejunum of nonhuman primates exposed to supralethal dose of total- or partial-body radiation. BMC Genom..

[25] Vellichirammal N.N., Sethi S., Pandey S., Singh J., Wise S.Y., Carpenter A.D., Fatanmi O.O., Guda C., Singh V.K. (2022). Lung transcriptome of nonhuman primates exposed to total- and partial-body irradiation. Mol. Ther.-Nucleic Acids.

[26] Li Y., Girgis M., Jayatilake M., Serebrenik A.A., Cheema A.K., Kaytor M.D., Singh V.K. (2022). Pharmacokinetic and metabolomic studies with a BIO 300 Oral Powder formulation in nonhuman primates. Sci. Rep..

[27] Girgis M., Li Y., Ma J., Sanda M., Wise S.Y., Fatanmi O.O., Kaytor M.D., Cheema A.K., Singh V.K. (2020). Comparative proteomic analysis of serum from nonhuman primates administered BIO 300: A promising radiation countermeasure. Sci. Rep..

[28] Serebrenik A.A., Verduyn C.W., Kaytor M.D. (2023). Safety, Pharmacokinetics, and Biomarkers of an Amorphous Solid Dispersion of Genistein, a Radioprotectant, in Healthy Volunteers. Clin. Pharmacol. Drug Dev..

[29] Xia C., Braunstein Z., Toomey A.C., Zhong J., Rao X. (2017). S100 Proteins As an Important Regulator of Macrophage Inflammation. Front. Immunol..

[30] Carr D.J. (1998). Increased levels of IFN-gamma in the trigeminal ganglion correlate with protection against HSV-1-induced encephalitis following subcutaneous administration with androstenediol. J. Neuroimmunol..

[31] Haroun R., Naasri S., Oweida A.J. (2023). Toll-Like Receptors and the Response to Radiotherapy in Solid Tumors: Challenges and Opportunities. Vaccines.

[32] U.S. Food and Drug Administration (2018). More about Biomarkers & Qualification. https://www.fda.gov/Drugs/DevelopmentApprovalProcess/DrugDevelopmentToolsQualificationProgram/BiomarkerQualificationProgram/ucm535408.htm.

[33] Ha C.T., Li X.-H., Fu D., Xiao M., Landauer M.R. (2013). Genistein nanoparticles protect mouse hematopoietic system and prevent proinflammatory factors after gamma irradiation. Radiat. Res..

[34] Jia Z., Babu P.V.A., Si H., Nallasamy P., Zhu H., Zhen W., Misra H.P., Li Y., Liu D. (2013). Genistein inhibits TNF-α-induced endothelial inflammation through the protein kinase pathway A and improves vascular inflammation in C57BL/6 mice. Int. J. Cardiol..

[35] Sutrisno S., Aprina H., Simanungkalit H.M., Andriyani A., Barlianto W., Sujuti H., Santoso S., Dwijayasa P.M., Wahyuni E.S., Mustofa E. (2018). Genistein modulates the estrogen receptor and suppresses angiogenesis and inflammation in the murine model of peritoneal endometriosis. J. Tradit. Complement. Med..

[36] U.S. Food and Drug Administration (2005). Guidance for Industry: Estimating the Maximum Safe Starting Dose in Initial Clinical Trials for Therapeutics in Adult Healthy Volunteers. https://www.fda.gov/regulatory-information/search-fda-guidance-documents/estimating-maximum-safe-starting-dose-initial-clinical-trials-therapeutics-adult-healthy-volunteers.

[37] Simone C.B., Serebrenik A.A., Gore E.M., Mohindra P., Brown S.L., Wang D., Chetty I.J., Vujaskovic Z., Menon S., Thompson J. (2023). Multicenter phase 1b/2a clinical trial of radioprotectant BIO 300 oral suspension for patients with non-small cell lung cancer receiving concurrent chemoradiotherapy. Int. J. Radiat. Oncol. Biol. Phys..

[38] Chen B.J., Deoliveira D., Spasojevic I., Sempowski G.D., Jiang C., Owzar K., Wang X., Gesty-Palmer D., Cline J.M., Bourland J.D. (2010). Growth hormone mitigates against lethal irradiation and enhances hematologic and immune recovery in mice and nonhuman primates. PLoS ONE.

[39] Jackson I.L., Zodda A., Gurung G., Pavlovic R., Kaytor M.D., Kuskowski M.A., Vujaskovic Z. (2017). BIO 300, a nanosuspension of genistein, mitigates pneumonitis/fibrosis following high-dose radiation exposure in the C57L/J murine model. Br. J. Pharmacol..

[40] Huang W., Yu J., Jones J.W., Carter C.L., Pierzchalski K., Tudor G., Booth C., MacVittie T.J., Kane M.A. (2019). Proteomic evaluation of the acute radiation syndrome of the gastrointestinal tract in a murine total-body irradiation model. Health Phys..

[41] Patterson A.M., Vemula S., Plett P.A., Sampson C.H., Chua H.L., Fisher A., Wu T., Sellamuthu R., Feng H., Katz B.P. (2022). Age and Sex Divergence in hematopoietic radiosensitivity in aged mouse models of the hematopoietic acute radiation syndrome. Radiat. Res..

[42] Li Y., Singh J., Varghese R., Zhang Y., Fatanmi O.O., Cheema A.K., Singh V.K. (2021). Transcriptome of rhesus macaque (*Macaca mulatta*) exposed to total-body irradiation. Sci. Rep..

[43] Bolger A.M., Lohse M., Usadel B. (2014). Trimmomatic: A flexible trimmer for Illumina sequence data. Bioinformatics.

[44] Dobin A., Davis C.A., Schlesinger F., Drenkow J., Zaleski C., Jha S., Batut P., Chaisson M., Gingeras T.R. (2013). STAR: Ultrafast universal RNA-seq aligner. Bioinformatics.

[45] Liao Y., Smyth G.K., Shi W. (2013). The Subread aligner: Fast, accurate and scalable read mapping by seed-and-vote. Nucleic Acids Res..

[46] Love M.I., Huber W., Anders S. (2014). Moderated estimation of fold change and dispersion for RNA-seq data with DESeq2. Genome Biol..

[47] Krämer A., Green J., Pollard J., Tugendreich S. (2014). Causal analysis approaches in Ingenuity Pathway Analysis. Bioinformatics.

