# Supplementary material for: BIO 300 Attenuates Whole Blood Transcriptome Changes in Mice Exposed to Total-Body Radiation
Source: Int J Mol Sci. 2024 Aug 13;25(16):8818. doi: 10.3390/ijms25168818 (PMC11354227; doi:10.3390/ijms25168818)
Supplement: Supplementary file 1 [file ijms-25-08818-s001.zip › ijms-3127309-supplementary.pdf]

SUPPLEMENTARY INFORMATION FOR:

BIO 300 attenuates whole blood transcriptome changes in mice exposed to total-body radiation

Artur A. Serebrenik, Oluseyi O. Fatanmi, Stephen Y. Wise, Sarah A Petrus, Michael D. Kaytor, Vijay K. Singh

TABLE OF CONTENTS:

Supplementary Figure S1. Top enriched signaling pathways in mice treated with a 50 mg/kg dose of BIO 300.

Supplementary Figure S2. Top enriched signaling pathways in mice treated with a 100 mg/kg dose of BIO 300.

Supplementary Table S1. Pathways for Figure 4A

Supplementary Table S2. Pathways for Figure 4B

Supplementary Table S3. S100 signaling pathway genes log2 fold change

Supplementary Table S4. Pathogen-induced cytokine storm signaling pathway genes log2 fold change

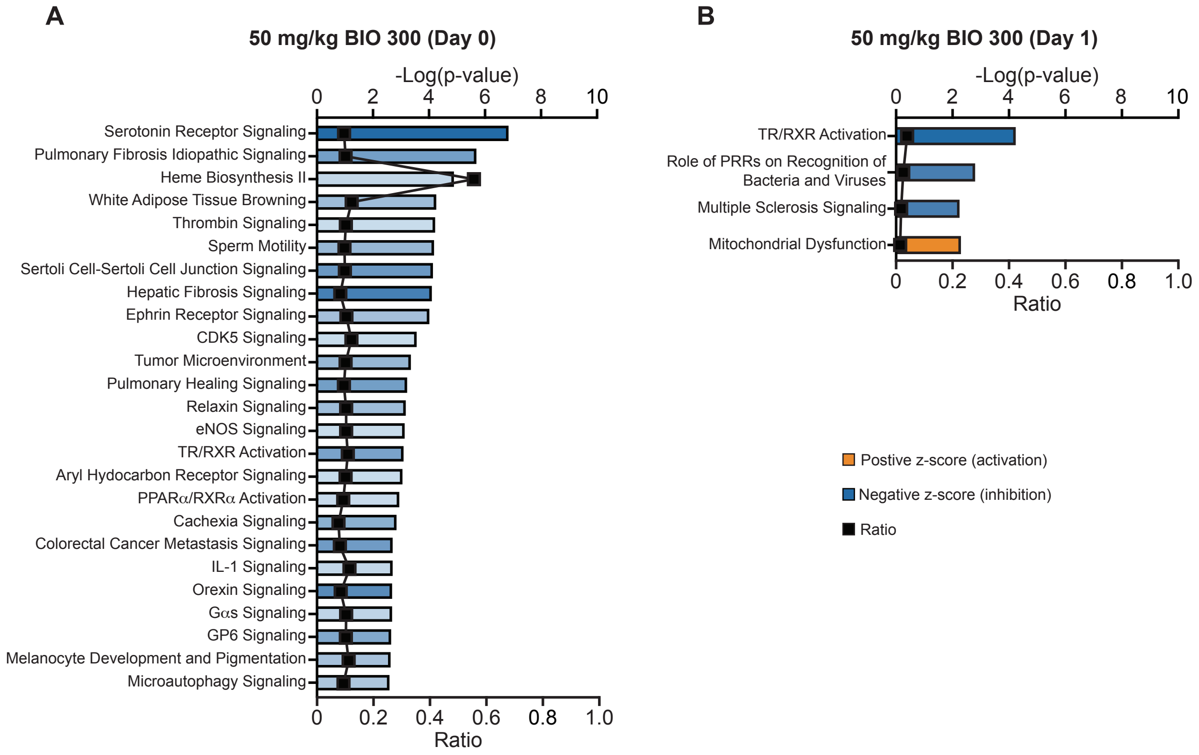

Supplementary Figure S1. Top enriched signaling pathways in mice treated with a 50 mg/kg dose of BIO 300. Top 25 canonical pathways identified using IPA that have a Fisher's exact test $p<0.05$ and an absolute z-score>2 in animals treated with BIO 300 (50 mg/kg, BID) at (**A**) 24 h (Day 0) and (**B**) 48 h (Day 1) after their last dose of BIO 300. Gene expression was compared to the respective vehicle groups in order to determine the top predicted activated or inhibited pathways. Blue bars indicate predicted inhibition of the pathway and orange bars indicate predicted activation. The darker the shading of the bars, the higher the absolute z-score. The black symbols represent the ratio of enriched genes in the data set compared to the total number of genes associated with the pathway. If less than 25 pathways are shown, then all enriched canonical pathways are represented.

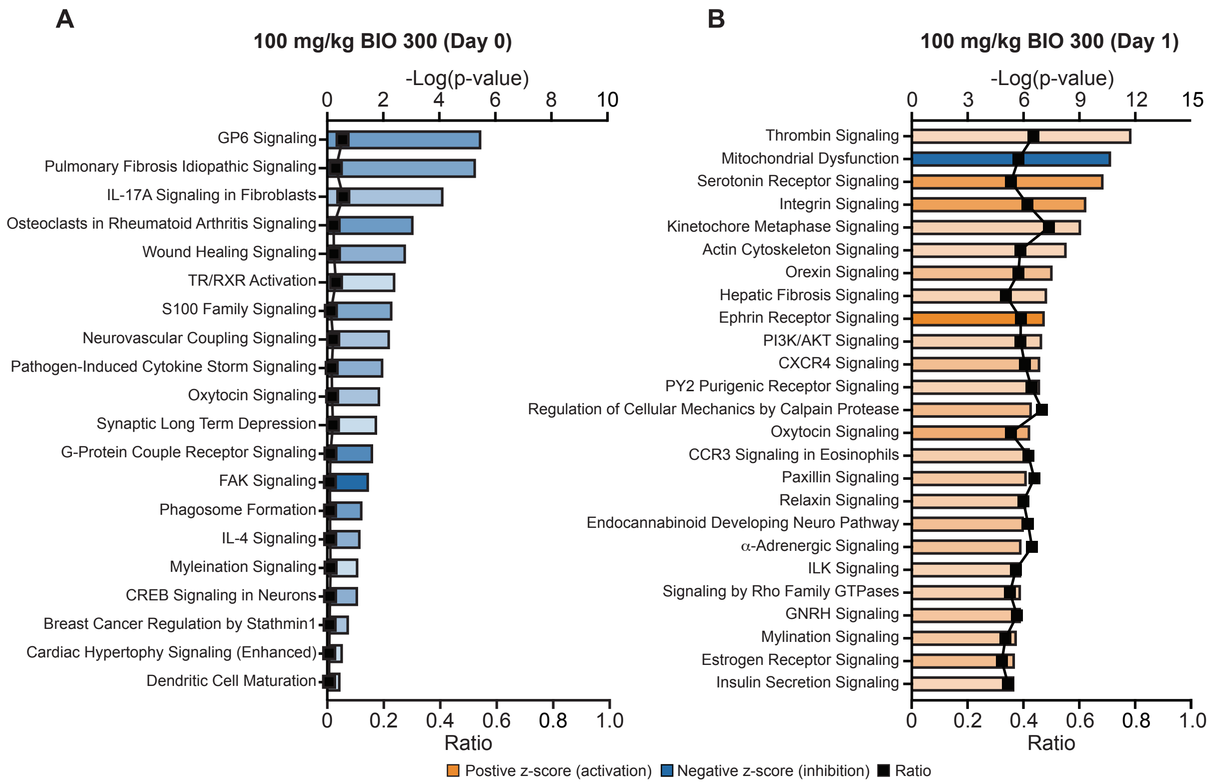

Supplementary Figure S2. Top enriched signaling pathways in mice treated with a 100 mg/kg dose of BIO 300. Top 25 canonical pathways identified using IPA that have a Fisher's exact test $p<0.05$ and an absolute z-score>2 in animals treated with BIO 300 (100 mg/kg, BID) at (**A**) 24 h (Day 0) and (**B**) 48 h (Day 1) after their last dose of BIO 300. Gene expression was compared to the respective vehicle groups in order to determine the top predicted activated or inhibited pathways. Blue bars indicate predicted inhibition of the pathway and orange bars indicate predicted activation. The darker the shading of the bars, the higher the absolute z-score. The black symbols represent the ratio of enriched genes in the data set compared to the total number of genes associated with the pathway. If less than 25 pathways are shown, then all enriched canonical pathways are represented.

Supplementary Table S1. Pathways for Figure 4A

© 2000-2024 QIAGEN. All rights reserved.				
**Ingenuity Canonical Pathway Category**	**Ingenuity Canonical Pathways**	**-log(p-value)**	**Ratio**	**z-score**
Cellular Growth, Proliferation and Development	DHCR24 Signaling Pathway	24.9	0.292	-6.008
Ingenuity Toxicity List Pathways, Nuclear Receptor Signaling	LXR/RXR Activation	28.1	0.333	-5.048
Cellular Stress and Injury, Disease-Specific Pathways	Pulmonary Fibrosis Idiopathic Signaling Pathway	3.15	0.0736	-4.491
Disease-Specific Pathways	Pathogen Induced Cytokine Storm Signaling Pathway	2.11	0.062	-4.379
Cellular Stress and Injury, Cancer	S100 Family Signaling Pathway	1.37	0.048	-4.11
Ingenuity Toxicity List Pathways, Xenobiotic Metabolism	Xenobiotic Metabolism PXR Signaling Pathway	6.86	0.123	-3.962
Cellular Growth, Proliferation and Development, Organismal Growth and Development	Human Embryonic Stem Cell Pluripotency	2.23	0.0746	-3.873
Ingenuity Toxicity List Pathways, Xenobiotic Metabolism	Xenobiotic Metabolism General Signaling Pathway	2.73	0.0909	-3.606
Cardiovascular Signaling	HIF1α Signaling	2.47	0.0769	-3.5
Cellular Stress and Injury	Wound Healing Signaling Pathway	1.71	0.0635	-3.5
Cancer	MSP-RON Signaling in Cancer Cells Pathway	2.35	0.0857	-3.464
Cellular Immune Response	Production of Nitric Oxide and Reactive Oxygen Species in Macrophages	1.38	0.0628	-3.464
Cytokine Signaling, Apoptosis, Cellular Immune Response	IL-15 Production	2.34	0.0894	-3.317
Cancer	Role of Tissue Factor in Cancer	3.32	0.087	-3.3
Ingenuity Toxicity List Pathways, Nuclear Receptor Signaling	TR/RXR Activation	8.28	0.164	-3.273
Ingenuity Toxicity List Pathways, Xenobiotic Metabolism	Xenobiotic Metabolism CAR Signaling Pathway	5.73	0.113	-3.273
Cardiovascular Signaling, Cellular Stress and Injury	Intrinsic Prothrombin Activation Pathway	12.4	0.381	-3.207
Cytokine Signaling	Role of JAK family kinases in IL-6-type Cytokine Signaling	3.31	0.127	-3.162
Cellular Growth, Proliferation and Development, Transcriptional Regulation	STAT3 Pathway	1.64	0.0741	-3
	GP6 Signaling Pathway	2.7	0.0945	-2.887
Cellular Growth, Proliferation and Development, Neurotransmitters and Other Nervous System Signaling	Myelination Signaling Pathway	1.33	0.055	-2.828
Cancer	Tumor Microenvironment Pathway	1.56	0.067	-2.714
Cytokine Signaling	IL-17A Signaling in Fibroblasts	1.44	0.0805	-2.646
Ingenuity Toxicity List Pathways, Xenobiotic Metabolism	Xenobiotic Metabolism AHR Signaling Pathway	2.97	0.115	-2.53
Ingenuity Toxicity List Pathways, Nuclear Receptor Signaling	RAR Activation	2.47	0.0629	-2.502
Cellular Stress and Injury, Cancer	Cachexia Signaling Pathway	1.62	0.0571	-2.4
Organismal Growth and Development	White Adipose Tissue Browning Pathway	2.4	0.087	-2.309
Ingenuity Toxicity List Pathways, Cytokine Signaling	Acute Phase Response Signaling	22.4	0.232	-2.236
Cancer, Disease-Specific Pathways	Estrogen-Dependent Breast Cancer Signaling	3.18	0.122	-2.236
Cellular Stress and Injury	Apelin Liver Signaling Pathway	2.62	0.185	-2.236
Organismal Growth and Development	Sperm Motility	1.95	0.0661	-2.236
Ingenuity Toxicity List Pathways, Nuclear Receptor Signaling	PXR/RXR Activation	10.1	0.262	-2.183
Cellular Stress and Injury, Disease-Specific Pathways	Type II Diabetes Mellitus Signaling	1.67	0.0719	-2

Supplementary Table S2. Pathways for Figure 4B

© 2000-2024 QIAGEN. All rights reserved.

Ingenuity Canonical Pathway Category	Ingenuity Canonical Pathways	-log(p-value)	Ratio	z-score
Apoptosis, Cell Cycle Regulation	14-3-3-mediated Signaling	3.74	0.394	3.667
Organismal Growth and Development, Cardiovascular Signaling, Cellular Stress and Injury	ABRA Signaling Pathway	3.13	0.402	4.11
Neurotransmitters and Other Nervous System Signaling	Acetylcholine Receptor Signaling Pathway	2.44	0.335	3.101
Organismal Growth and Development	Actin Cytoskeleton Signaling	7.16	0.402	4.431
Intracellular and Second Messenger Signaling	Actin Nucleation by ARP-WASP Complex	8.74	0.538	3.772
Organismal Growth and Development	Activin Inhibin Signaling Pathway	7.4	0.415	4.006
Disease-Specific Pathways, Cancer	Acute Myeloid Leukemia Signaling	5.59	0.473	2.197
Ingenuity Toxicity List Pathways, Cytokine Signaling	Acute Phase Response Signaling	16.4	0.535	5.416
Intracellular and Second Messenger Signaling, Neurotransmitters and Other Nervous System Signaling	Adrenergic Receptor Signaling Pathway (Enhanced)	2.7	0.34	3.395
Cellular Stress and Injury, Cellular Growth, Proliferation and Development	Adrenomedullin signaling pathway	6	0.402	5.657
Neurotransmitters and Other Nervous System Signaling	Agrin Interactions at Neuromuscular Junction	5.05	0.493	3.9
Intracellular and Second Messenger Signaling, Cellular Growth, Proliferation and Development	AMPK Signaling	4.99	0.372	4.989
Cardiovascular Signaling, Cellular Growth, Proliferation and Development	Angiopoietin Signaling	2.5	0.395	3.674
Cellular Stress and Injury	Antioxidant Action of Vitamin C	3.78	0.404	-3.888
Cell Cycle Regulation	Antiproliferative Role of Somatostatin Receptor 2	3.43	0.429	3.3
Intracellular and Second Messenger Signaling	Apelin Adipocyte Signaling Pathway	6.05	0.484	2.271
Cardiovascular Signaling, Cellular Stress and Injury	Apelin Cardiomyocyte Signaling Pathway	2.19	0.364	3.333
Organismal Growth and Development	Apelin Endothelial Signaling Pathway	5.97	0.433	3.92
Cellular Stress and Injury	Apelin Liver Signaling Pathway	1.7	0.444	2.887
Intracellular and Second Messenger Signaling	Apelin Muscle Signaling Pathway	6.14	0.583	3.317
Cellular Stress and Injury, Organismal Growth and Development	Autophagy	2.14	0.323	2.216
Humoral Immune Response	B Cell Receptor Signaling	8.25	0.348	3.2
Disease-Specific Pathways, Cancer	Basal Cell Carcinoma Signaling	1.69	0.361	2.065
Disease-Specific Pathways, Cancer	Bladder Cancer Signaling	4.96	0.431	2.357
Organismal Growth and Development	BMP signaling pathway	3.95	0.429	3.307
Disease-Specific Pathways, Cancer	Breast Cancer Regulation by Stathmin1	5.19	0.327	6.249
Cellular Stress and Injury	Cachexia Signaling Pathway	2.59	0.312	5.502
Intracellular and Second Messenger Signaling	cAMP-mediated signaling	5.79	0.386	3.622
Xenobiotic Metabolism, Cancer, Ingenuity Toxicity List Pathways	Cancer Drug Resistance by Drug Efflux	2.41	0.414	3.545
Cardiovascular Signaling, Disease-Specific Pathways	Cardiac Hypertrophy Signaling	7.99	0.406	6.037
Organismal Growth and Development, Cardiovascular Signaling	Cardiac Hypertrophy Signaling (Enhanced)	10.1	0.371	7.653
Cardiovascular Signaling	Cardiac β-adrenergic Signaling	2.05	0.328	2.921
Cellular Immune Response, Cytokine Signaling	CCR3 Signaling in Eosinophils	4.98	0.418	4.271
Organismal Growth and Development, Cytokine Signaling	Chemokine Signaling	5.38	0.481	3.452
Neurotransmitters and Other Nervous System Signaling	Cholecystokinin/Gastrin-mediated Signaling	7.45	0.479	4.901
Disease-Specific Pathways, Cancer	Chronic Myeloid Leukemia Signaling	6.07	0.378	4.001
Transcriptional Regulation, Cellular Stress and Injury	CLEAR Signaling Pathway	8.74	0.407	-2.248
Disease-Specific Pathways, Cancer	Colorectal Cancer Metastasis Signaling	15.7	0.476	6
Humoral Immune Response	Complement System	13.2	0.838	2.065
Disease-Specific Pathways	Coronavirus Pathogenesis Pathway	7.13	0.417	3.183
Cellular Growth, Proliferation and Development	Corticotropin Releasing Hormone Signaling	3.51	0.375	4.323
Cellular Growth, Proliferation and Development	CREB Signaling in Neurons	7.42	0.344	6.947
Cellular Immune Response, Cytokine Signaling	CXCR4 Signaling	10.5	0.482	4.225
Cellular Growth, Proliferation and Development	DHCR24 Signaling Pathway	13.9	0.555	6.882
Growth Factor Signaling, Cellular Growth, Proliferation and Development	EGF Signaling	1.64	0.375	2.683
Intracellular and Second Messenger Signaling	Eicosanoid Signaling	4.41	0.471	3.638
Growth Factor Signaling, Organismal Growth and Development, Neurotransmitters and Other Nervous System Signaling	Endocannabinoid Developing Neuron Pathway	7.09	0.465	3.618
Disease-Specific Pathways, Cancer	Endometrial Cancer Signaling	3.74	0.467	3.128
Cardiovascular Signaling	Endothelin-1 Signaling	8.35	0.438	5.208
Cardiovascular Signaling	eNOS Signaling	2.02	0.333	4.003
Organismal Growth and Development, Neurotransmitters and Other Nervous System Signaling	Ephrin B Signaling	7.66	0.556	4.017
Organismal Growth and Development, Neurotransmitters and Other Nervous System Signaling	Ephrin Receptor Signaling	13.7	0.495	6.647
Growth Factor Signaling, Neurotransmitters and Other Nervous System Signaling	ERB2-ERBB3 Signaling	2.67	0.415	3.4
Growth Factor Signaling, Neurotransmitters and Other Nervous System Signaling	ERBB Signaling	3.02	0.398	3.773
Growth Factor Signaling	ERBB4 Signaling	2.35	0.397	2.985
Intracellular and Second Messenger Signaling, Cancer	ERK/MAPK Signaling	4.79	0.377	4.158
Intracellular and Second Messenger Signaling	ERK5 Signaling	4.68	0.473	3.772
Growth Factor Signaling	Erythropoietin Signaling Pathway	1.88	0.324	2.94
Nuclear Receptor Signaling	Estrogen Receptor Signaling	4.24	0.333	7.056
Cellular Growth, Proliferation and Development, Disease-Specific Pathways, Cancer	Estrogen-Dependent Breast Cancer Signaling	6.67	0.512	4.914
Organismal Growth and Development, Cardiovascular Signaling	Factors Promoting Cardiogenesis in Vertebrates	3.16	0.366	4.16
Cancer	FAK Signaling	1.54	0.273	4.984
Humoral Immune Response	Fc Epsilon RI Signaling	2.79	0.373	3.395
Cellular Immune Response	Fcγ Receptor-mediated Phagocytosis in Macrophages and Monocytes	5.14	0.457	4.727
Apoptosis, Cellular Stress and Injury	Ferroptosis Signaling Pathway	5.72	0.435	2.562
Growth Factor Signaling, Cellular Growth, Proliferation and Development	FGF Signaling	4.2	0.442	4.226
Cytokine Signaling	FLT3 Signaling in Hematopoietic Progenitor Cells	4.35	0.451	3.656
Cellular Immune Response, Cytokine Signaling	fMLP Signaling in Neutrophils	4.29	0.405	3.667
Intracellular and Second Messenger Signaling	G Beta Gamma Signaling	4.85	0.419	4.33
Intracellular and Second Messenger Signaling	G-Protein Coupled Receptor Signaling	8.69	0.346	6.119
Neurotransmitters and Other Nervous System Signaling	GABAergic Receptor Signaling Pathway (Enhanced)	1.41	0.317	2.412
Growth Factor Signaling, Neurotransmitters and Other Nervous System Signaling	GDNF Family Ligand-Receptor Interactions	2.19	0.382	3.8
Disease-Specific Pathways, Cancer	Glioblastoma Multiforme Signaling	9.1	0.462	5.17
Disease-Specific Pathways, Cancer	Glioma Invasiveness Signaling	9.27	0.589	4.11
Disease-Specific Pathways, Cancer	Glioma Signaling	5.72	0.44	4.116
Neurotransmitters and Other Nervous System Signaling	Glutaminergic Receptor Signaling Pathway (Enhanced)	3.89	0.338	5.149
Growth Factor Signaling, Cellular Immune Response, Cellular Growth, Proliferation and Development, Cytokine Signaling	GM-CSF Signaling	3.98	0.457	3.528
Neurotransmitters and Other Nervous System Signaling	GNRH Signaling	5.22	0.393	5.443
Cellular Stress and Injury	GP6 Signaling Pathway	5.46	0.433	5.741
Growth Factor Signaling, Cellular Growth, Proliferation and Development	Growth Hormone Signaling	1.51	0.352	2.683
Intracellular and Second Messenger Signaling	Gα12/13 Signaling	2.91	0.368	3.539
Intracellular and Second Messenger Signaling	Gαi Signaling	10.5	0.507	2.06
Intracellular and Second Messenger Signaling	Gαq Signaling	8.83	0.459	3.207
Intracellular and Second Messenger Signaling	Gαs Signaling	4.85	0.421	4.004
Cellular Stress and Injury, Cellular Growth, Proliferation and Development, Disease-Specific Pathways	Hepatic Fibrosis Signaling Pathway	15	0.423	7.568
Disease-Specific Pathways, Cancer	HER-2 Signaling in Breast Cancer	4.35	0.366	2.556
Growth Factor Signaling, Organismal Growth and Development, Cellular Growth, Proliferation and Development	HGF Signaling	7.2	0.462	5.032
Cardiovascular Signaling, Cellular Stress and Injury	HIF1α Signaling	6.36	0.404	5.598
Humoral Immune Response, Cellular Immune Response, Cellular Stress and Injury, Cytokine Signaling	HMGB1 Signaling	3.9	0.377	3.539
Cancer	HOTAIR Regulatory Pathway	2.22	0.337	2.774
Organismal Growth and Development, Cellular Growth, Proliferation and Development	Human Embryonic Stem Cell Pluripotency	3.08	0.348	5.976
Transcriptional Regulation, Organismal Growth and Development, Cancer	ID1 Signaling Pathway	7.84	0.428	4.745
Growth Factor Signaling, Cellular Growth, Proliferation and Development	IGF-1 Signaling	5.23	0.448	4.596
Cytokine Signaling	IL-1 Signaling	6.62	0.49	2.837
Cellular Immune Response, Cytokine Signaling	IL-10 Signaling	6.59	0.435	2.321
Cytokine Signaling	IL-13 Signaling Pathway	2.42	0.362	4.32
Cellular Immune Response, Cytokine Signaling	IL-15 Production	8.18	0.488	5.077
Apoptosis, Cellular Immune Response, Cytokine Signaling	IL-15 Signaling	5.55	0.335	2.785
Pathogen-Influenced Signaling, Cellular Immune Response, Cytokine Signaling	IL-17 Signaling	1.37	0.305	4.371
Cytokine Signaling	IL-17A Signaling in Fibroblasts	4.47	0.448	4.003
Cytokine Signaling	IL-17A Signaling in Gastric Cells	1.85	0.462	2.333
Cellular Immune Response, Cytokine Signaling	IL-2 Signaling	1.99	0.387	3.411
Cytokine Signaling	IL-3 Signaling	2.21	0.38	3.9
Cellular Immune Response, Cytokine Signaling	IL-33 Signaling Pathway	1.97	0.324	4.901
Cellular Immune Response, Cytokine Signaling	IL-6 Signaling	4.17	0.403	5.105
Cellular Immune Response, Cytokine Signaling	IL-8 Signaling	12.8	0.481	6.915
Cellular Growth, Proliferation and Development	ILK Signaling	12	0.478	4.756
Cardiovascular Signaling	Inhibition of Angiogenesis by TSP1	6.02	0.647	2.5
Cellular Immune Response	iNOS Signaling	2.65	0.447	2
Intracellular and Second Messenger Signaling	Insulin Receptor Signaling	2.38	0.35	2.534
Organismal Growth and Development	Insulin Secretion Signaling Pathway	2.98	0.331	5.128
Intracellular and Second Messenger Signaling, Cell Cycle Regulation, Hemostasis, Cellular Growth, Proliferation and Development	Integrin Signaling	10.7	0.458	6.183
Cardiovascular Signaling, Cellular Stress and Injury	Intrinsic Prothrombin Activation Pathway	5.13	0.571	2.985
Apoptosis, Intracellular and Second Messenger Signaling, Cellular Growth, Proliferation and Development	JAK/STAT Signaling	3.56	0.427	2.611

Disease-Specific Pathways	Leptin Signaling in Obesity	3.56	0.434	2.84
Cellular Immune Response	Leukocyte Extravasation Signaling	10.5	0.466	3.349
Apoptosis, Pathogen-Influenced Signaling	LPS-stimulated MAPK Signaling	3.21	0.412	3.651
Nuclear Receptor Signaling, Ingenuity Toxicity List Pathways	LXR/RXR Activation	16.2	0.602	3
Cellular Immune Response, Organismal Growth and Development	Macropinocytosis Signaling	5.28	0.487	3.545
Growth Factor Signaling, Cellular Growth, Proliferation and Development	Melanocyte Development and Pigmentation Signaling	4.58	0.439	5
Disease-Specific Pathways, Cancer	Melanoma Signaling	3.07	0.46	3.273
Cellular Immune Response	MIF Regulation of Innate Immunity	2.25	0.432	2.828
Cellular Immune Response, Nuclear Receptor Signaling	MIF-mediated Glucocorticoid Regulation	1.72	0.417	2.673
Organismal Growth and Development, Cellular Growth, Proliferation and Development	Mouse Embryonic Stem Cell Pluripotency	5.37	0.452	4.025
Cancer	MSP-RON Signaling in Cancer Cells Pathway	9.55	0.493	5.085
Pathogen-Influenced Signaling, Cellular Immune Response, Cellular Stress and Injury	MSP-RON Signaling in Macrophages Pathway	6.14	0.454	4.808
Cellular Growth, Proliferation and Development	mTOR Signaling	5.76	0.393	3.063
Disease-Specific Pathways	Multiple Sclerosis Signaling Pathway	5.03	0.378	3.273
Apoptosis	MYC Mediated Apoptosis Signaling	1.59	0.38	2.065
Cellular Growth, Proliferation and Development, Neurotransmitters and Other Nervous System Signaling	Myelination Signaling Pathway	13.1	0.434	6.042
Intracellular and Second Messenger Signaling	NAD Signaling Pathway	2.8	0.358	4.341
Cellular Immune Response	Natural Killer Cell Signaling	5.47	0.394	2.065
Growth Factor Signaling, Neurotransmitters and Other Nervous System Signaling	Neuregulin Signaling	6.01	0.453	3.43
Neurotransmitters and Other Nervous System Signaling	Neuroinflammation Signaling Pathway	6.62	0.375	4.159
Neurotransmitters and Other Nervous System Signaling	Neurotrophin/TRK Signaling	4.51	0.462	3.413
Cellular Stress and Injury	Neurovascular Coupling Signaling Pathway	2.28	0.323	5.812
Cellular Immune Response, Cellular Stress and Injury	Neutrophil Extracellular Trap Signaling Pathway	2.5	0.308	3.697
Pathogen-Influenced Signaling, Cellular Immune Response	NF-κB Activation by Viruses	3.31	0.423	2.694
Growth Factor Signaling, Neurotransmitters and Other Nervous System Signaling	NGF Signaling	2.9	0.375	4.427
Cardiovascular Signaling	Nitric Oxide Signaling in the Cardiovascular System	2.12	0.35	3.773
Disease-Specific Pathways, Cancer	Non-Small Cell Lung Cancer Signaling	4.72	0.447	3.024
Cellular Stress and Injury, Ingenuity Toxicity List Pathways	NRF2-mediated Oxidative Stress Response	7.91	0.414	4.964
Cellular Growth, Proliferation and Development, Cytokine Signaling	Oncostatin M Signaling	2.39	0.442	3.638
Neurotransmitters and Other Nervous System Signaling	Opioid Signaling Pathway	8.61	0.407	2.65
Neurotransmitters and Other Nervous System Signaling	Orexin Signaling Pathway	4.27	0.361	5.176
Cellular Stress and Injury, Disease-Specific Pathways	Osteoarthritis Pathway	10.6	0.445	4.076
Disease-Specific Pathways, Cancer	Ovarian Cancer Signaling	5.06	0.405	4.49
Neurotransmitters and Other Nervous System Signaling	Oxytocin in Brain Signaling Pathway	4.49	0.377	4.007
Organismal Growth and Development	Oxytocin Signaling Pathway	7.15	0.39	6.928
Other Pathways	P2Y Purigenic Receptor Signaling Pathway	7.92	0.474	4.429
Intracellular and Second Messenger Signaling, Humoral Immune Response, Cellular Immune Response, Cellular Stress and Injury, Cytokine Signaling	p38 MAPK Signaling	3.19	0.383	4.427
Cellular Stress and Injury, Cellular Growth, Proliferation and Development	p70S6K Signaling	7.12	0.344	3.753
Intracellular and Second Messenger Signaling	PAK Signaling	6.43	0.462	4.439
Disease-Specific Pathways, Cancer	Pancreatic Adenocarcinoma Signaling	3.84	0.397	4.116
Disease-Specific Pathways	Pathogen Induced Cytokine Storm Signaling Pathway	9.88	0.396	5.526
Organismal Growth and Development	Paxillin Signaling	8.09	0.505	4.644
Cellular Growth, Proliferation and Development	PDGF Signaling	2.67	0.391	4.352
Cellular Growth, Proliferation and Development	PEDF Signaling	4.08	0.44	3.307
Cellular Immune Response, Cancer	PFKFB4 Signaling Pathway	4.38	0.521	3.411
Pathogen-Influenced Signaling, Cellular Immune Response	Phagosome Formation	11.4	0.363	8.222
Intracellular and Second Messenger Signaling	Phospholipase C Signaling	3.01	0.287	3.411
Cellular Immune Response	PI3K Signaling in B Lymphocytes	9.43	0.36	2.292
Intracellular and Second Messenger Signaling, Cellular Growth, Proliferation and Development, Cancer	PI3K/AKT Signaling	7.24	0.42	2.967
Nuclear Receptor Signaling	PPAR Signaling	4.21	0.421	-4.117
Cellular Immune Response	Production of Nitric Oxide and Reactive Oxygen Species in Macrophages	5.85	0.403	4.366
Organismal Growth and Development, Cytokine Signaling	Prolactin Signaling	1.73	0.347	3.024
Apoptosis, Cancer	PTEN Signaling	10	0.49	-4.296
Cellular Stress and Injury, Disease-Specific Pathways	Pulmonary Fibrosis Idiopathic Signaling Pathway	14.4	0.445	8.727
Cellular Stress and Injury	Pulmonary Healing Signaling Pathway	10.9	0.467	5.911
Nuclear Receptor Signaling, Ingenuity Toxicity List Pathways	PXR/RXR Activation	8.14	0.585	3.893
Intracellular and Second Messenger Signaling	RAC Signaling	4	0.394	5.516
Cellular Growth, Proliferation and Development, Disease-Specific Pathways	RANK Signaling in Osteoclasts	2.03	0.363	3.9
Nuclear Receptor Signaling, Ingenuity Toxicity List Pathways	RAR Activation	7.13	0.361	6.667
Organismal Growth and Development, Neurotransmitters and Other Nervous System Signaling	Reelin Signaling in Neurons	4.88	0.413	4.128
Neurotransmitters and Other Nervous System Signaling	Regulation of Actin-based Motility by Rho	4.35	0.417	2.785
Cellular Growth, Proliferation and Development	Regulation of Cellular Mechanics by Calpain Protease	7.21	0.511	3.273
Cellular Stress and Injury, Cellular Growth, Proliferation and Development	Regulation of eIF4 and p70S6K Signaling	4.98	0.391	3.962
Growth Factor Signaling, Organismal Growth and Development	Regulation of the Epithelial Mesenchymal Transition by Growth Factors Pathw	4.54	0.38	5.093
Growth Factor Signaling, Organismal Growth and Development	Relaxin Signaling	6.1	0.426	5.048
Disease-Specific Pathways, Cancer	Renal Cell Carcinoma Signaling	2.52	0.392	4.2
Growth Factor Signaling, Cardiovascular Signaling	Renin-Angiotensin Signaling	3.7	0.397	3.781
Intracellular and Second Messenger Signaling	RHOA Signaling	3.72	0.395	3.92
Intracellular and Second Messenger Signaling	RHOGDI Signaling	8.11	0.423	-4.533
Cellular Growth, Proliferation and Development	Ribonucleotide Reductase Signaling Pathway	3.92	0.376	2.5
Disease-Specific Pathways	Role of Chondrocytes in Rheumatoid Arthritis Signaling Pathway	8.01	0.468	5.416
Pathogen-Influenced Signaling, Disease-Specific Pathways	Role of Hypercytokinemia/hyperchemokinemia in the Pathogenesis of Influenz	1.42	0.337	3.9
Cytokine Signaling	Role of JAK family kinases in IL-6-type Cytokine Signaling	5.72	0.494	3.363
Cytokine Signaling	Role of JAK2 in Hormone-like Cytokine Signaling	3.88	0.468	3.78
Pathogen-Influenced Signaling, Disease-Specific Pathways	Role of MAPK Signaling in Inhibiting the Pathogenesis of Influenza	3.95	0.443	4.352
Pathogen-Influenced Signaling, Disease-Specific Pathways	Role of MAPK Signaling in Promoting the Pathogenesis of Influenza	4.11	0.412	5.729
Transcriptional Regulation, Organismal Growth and Development	Role of NANOG in Mammalian Embryonic Stem Cell Pluripotency	2.84	0.371	3.962
Cardiovascular Signaling, Disease-Specific Pathways, Cancer	Role of NFAT in Cardiac Hypertrophy	4.59	0.371	3.447
Disease-Specific Pathways	Role of Osteoclasts in Rheumatoid Arthritis Signaling Pathway	12.1	0.432	6.938
Pathogen-Influenced Signaling, Cellular Immune Response	Role of Pattern Recognition Receptors in Recognition of Bacteria and Viruses	2.93	0.359	3.55
Pathogen-Influenced Signaling, Cellular Immune Response	Role of PKR in Interferon Induction and Antiviral Response	3.21	0.375	3.479
Cancer	Role of Tissue Factor in Cancer	10.6	0.459	7.49
Cellular Immune Response	S100 Family Signaling Pathway	6.05	0.323	7.034
Organismal Growth and Development, Cellular Stress and Injury, Cellular Growth, Proliferation and Development	Senescence Pathway	2.15	0.311	3.579
Neurotransmitters and Other Nervous System Signaling	Serotonin Receptor Signaling	17.5	0.429	9.099
Cellular Growth, Proliferation and Development	Sertoli Cell-Sertoli Cell Junction Signaling	10.5	0.44	7.831
Intracellular and Second Messenger Signaling	Signaling by Rho Family GTPases	9.02	0.416	6.203
Organismal Growth and Development, Neurotransmitters and Other Nervous System Signaling	SNARE Signaling Pathway	1.76	0.331	3.727
Organismal Growth and Development	Sperm Motility	13.4	0.463	4.422
Intracellular and Second Messenger Signaling	Sphingosine-1-phosphate Signaling	8.22	0.492	2.828
Transcriptional Regulation, Cellular Growth, Proliferation and Development	STAT3 Pathway	14.3	0.563	4.389
Neurotransmitters and Other Nervous System Signaling	Synaptic Long Term Depression	3.05	0.348	4.914
Neurotransmitters and Other Nervous System Signaling	Synaptic Long Term Potentiation	2.01	0.341	2.03
Organismal Growth and Development, Neurotransmitters and Other Nervous System Signaling	Synaptogenesis Signaling Pathway	4.08	0.343	6.734
Cellular Immune Response, Disease-Specific Pathways	Systemic Lupus Erythematosus in B Cell Signaling Pathway	7.37	0.335	2.538
Apoptosis, Cancer	Telomerase Signaling	1.36	0.324	3.138
Growth Factor Signaling, Cellular Growth, Proliferation and Development, Ingenuity Toxicity List Pathways	TGF-β Signaling	4.46	0.438	3.212
Cardiovascular Signaling	Thrombin Signaling	14	0.484	4.007
Cellular Growth, Proliferation and Development	Thrombopoietin Signaling	2.92	0.429	3.53
Disease-Specific Pathways, Cancer	Thyroid Cancer Signaling	4.79	0.468	3.124
Apoptosis, Pathogen-Influenced Signaling, Humoral Immune Response, Cellular Immune Response	Toll-like Receptor Signaling	5.41	0.487	3.922
Nuclear Receptor Signaling, Ingenuity Toxicity List Pathways	TR/RXR Activation	2.78	0.367	5.397
Transcriptional Regulation, Organismal Growth and Development, Cellular Growth, Proliferation and Development	Transcriptional Regulatory Network in Embryonic Stem Cells	3.33	0.366	4.131
Cellular Immune Response, Cytokine Signaling	TREM1 Signaling	6.6	0.519	3.042
Cancer	Tumor Microenvironment Pathway	8.38	0.447	6.114
Cellular Stress and Injury	UVA-Induced MAPK Signaling	3.49	0.408	3.674
Cellular Stress and Injury	UVC-Induced MAPK Signaling	3.82	0.49	2.985
Growth Factor Signaling, Cellular Growth, Proliferation and Development	VEGF Family Ligand-Receptor Interactions	2.98	0.405	4.596
Growth Factor Signaling	VEGF Signaling	5.25	0.455	3.904
Cellular Growth, Proliferation and Development	Vitamin-C Transport	1.87	0.478	2.828
Organismal Growth and Development	White Adipose Tissue Browning Pathway	7.59	0.464	4.99
Organismal Growth and Development, Cancer	WNT/Ca+ pathway	2.56	0.409	2.858
Cellular Stress and Injury	Wound Healing Signaling Pathway	6.68	0.393	6.332
Xenobiotic Metabolism	Xenobiotic Metabolism AHR Signaling Pathway	4.47	0.448	5.191
Xenobiotic Metabolism, Ingenuity Toxicity List Pathways	Xenobiotic Metabolism CAR Signaling Pathway	4.65	0.381	5.578
Xenobiotic Metabolism, Ingenuity Toxicity List Pathways	Xenobiotic Metabolism General Signaling Pathway	7.7	0.462	6.574
Xenobiotic Metabolism, Ingenuity Toxicity List Pathways	Xenobiotic Metabolism PXR Signaling Pathway	5.14	0.39	7.14
	α-Adrenergic Signaling	7.73	0.495	2.887

Supplementary Table S3. S100 signaling pathway genes log2 fold change

Genes in the S100 Family Signaling Pathway network	Veh (Day 0) vs Veh (Day 1)	Veh (Day 1) vs Veh+TBI (Day 1)	Veh (Day 1) vs Veh+TBI (Day 1)	Veh (Day 0) vs BIO 300 200 mg/kg (Day 0)	Veh (Day 1) vs BIO 300 200 mg/kg (Day 1)	Veh+TBI (Day 1) vs BIO 300 200 mg/kg (Day 1)
ABCB1	-0.184	0.329	0.494	-0.212	-0.206	-0.158
ABCC1	-0.009	-0.244	-0.242	-0.012	0.583	0.402
ACKR1	-0.652	0.125	0.768	0.612	2.495	0.166
ACKR3	-0.353	2.552	2.888	-1.044	0.329	-0.592
ACKR4	-0.909	0.313	1.192	-0.774	N/A	-0.865
ADAM10	-0.251	1.159	1.401	0.523	2.177	0.030
ADGRA2	-0.019	-0.329	-0.321	-0.235	-1.705	-0.272
ADGRA3	-1.066	1.442	2.481	-0.415	1.873	-0.392
ADGRB1	N/A	N/A	N/A	N/A	N/A	0.307
ADGRB2	0.352	-1.089	-1.460	0.328	-1.856	0.384
ADGRD1	-0.822	1.506	2.339	-1.193	N/A	-0.644
ADGRE1	0.072	2.452	2.370	-0.180	-0.228	-0.803
Adgre4	0.472	-0.010	-0.491	-0.001	-0.494	-0.293
ADGRE5	0.003	1.352	1.335	-0.137	-0.204	-0.057
ADGRF5	-1.707	1.697	3.373	-1.597	0.444	0.132
ADGRG1	-0.611	2.154	2.756	0.259	3.154	0.368
ADGRG3	-0.075	1.886	1.947	-0.029	0.777	-0.134
ADGRG5	0.522	-1.815	-2.356	0.049	-1.406	0.100
ADGRG6	-0.003	1.620	1.608	-0.371	-0.210	-0.580
ADGRL1	0.049	-1.456	-1.504	-0.137	-1.498	0.847
ADGRL4	-0.746	1.871	2.589	-0.958	-0.065	-0.285
ADGRV1	N/A	2.104	1.987	N/A	N/A	-0.541
ADORA1	-1.259	1.827	3.045	-0.984	-0.218	-1.045
ADORA2A	-0.013	-0.601	-0.598	0.019	0.129	0.443
ADORA2B	0.469	-0.371	-0.867	-0.034	-0.799	-0.236
ADORA3	0.029	1.193	1.155	-0.211	0.400	-0.251
ADRA1A	-1.763	1.252	2.989	N/A	N/A	0.381
ADRA1B	-1.996	2.619	4.590	-1.578	0.501	-1.575
ADRA1D	N/A	N/A	N/A	N/A	N/A	-0.036
ADRA2A	N/A	2.369	2.795	N/A	2.826	0.262
ADRB1	0.125	0.409	0.269	0.263	0.599	1.107
ADRB2	0.127	1.699	1.555	-0.130	-0.966	-0.182
ADRB3	-1.332	1.798	3.105	-1.506	0.462	-1.154
AGER	0.302	1.779	1.440	0.242	-0.795	-0.038
AGTR1	-0.500	1.424	1.914	0.365	2.534	-0.154
AHNAK	-0.111	-0.695	-0.590	-0.346	-1.004	0.152
AKT1	0.054	1.120	1.051	-0.120	-0.108	-0.185
AKT2	-0.399	0.332	0.718	0.263	1.421	0.242
AKT3	-0.114	1.082	1.188	0.250	1.964	0.332
ANXA1	0.053	2.255	2.178	-0.246	0.572	-0.239
ANXA2	-0.008	1.899	1.887	-0.079	0.612	-0.188
APP	-0.230	2.283	2.490	-0.467	-0.616	-0.694
ARAF	-0.029	0.063	0.076	-0.129	-0.599	-0.137
ARNT	-0.119	0.165	0.272	-0.109	-0.103	-0.048
ATF2	-0.067	1.533	1.590	0.326	1.849	-0.075
ATF4	-0.134	1.327	1.449	0.193	1.837	0.265
ATP2A2	-0.110	0.407	0.502	-0.012	0.572	2.139
ATP5F1A	-0.301	-0.161	0.125	-0.079	0.413	0.500
ATP5F1B	-0.539	-0.514	0.014	0.446	2.054	0.291
ATP5F1C	0.075	-0.146	-0.237	-0.047	-0.022	0.622
ATP5F1D	-0.239	0.000	0.223	-0.304	0.077	0.147
Atp5f1e	-0.610	1.267	1.866	0.286	2.868	0.636
ATP5PO	-0.075	-0.060	-0.004	-0.110	0.074	0.649
AVPR1A	N/A	1.890	3.262	N/A	N/A	-0.655
AVPR2	0.185	-0.099	-0.308	-0.393	-2.277	-0.370
BCL2	0.103	-0.672	-0.784	0.001	-1.520	0.311
BCL3	-0.147	0.077	0.204	-0.137	-0.557	-0.236
BDNF	N/A	N/A	N/A	N/A	N/A	-0.630
BRAF	-0.147	0.507	0.642	0.077	0.569	-0.153
BSG	-0.605	0.431	1.024	0.555	2.284	0.201
C3AR1	-0.133	1.616	1.746	-0.181	0.448	-0.333
C5AR1	-0.079	3.113	3.172	-0.194	-0.144	-0.223
C5AR2	-0.085	2.970	3.036	-0.128	0.107	-0.270
CACNA1A	0.007	0.877	0.855	-0.220	-2.434	-0.467
CACNA1C	-0.135	0.420	0.529	0.135	1.953	3.238
CACNA1D	-0.052	-1.152	-1.106	-0.149	-1.925	-0.707
CACNA1E	0.087	-0.544	-0.645	0.016	-0.310	-0.180
CACNA1G	-0.518	0.999	1.512	0.480	2.617	0.232
CACNA1H	-0.168	0.371	0.538	0.271	1.374	0.131
CACNA1I	0.033	-4.435	-4.475	-0.201	-2.154	-0.099
CACNA2D1	0.412	2.261	1.820	N/A	2.225	2.495
CACNA2D2	0.256	-1.321	-1.618	0.095	-0.991	0.920
CACNA2D4	0.343	-0.851	-1.219	0.069	-2.386	-0.100
CACNB1	0.082	-2.133	-2.218	0.060	-2.143	0.458
CACNB2	-0.098	0.489	0.571	0.178	-0.059	1.942
CACNB3	-0.055	1.848	1.897	0.170	1.846	0.499
CACNB4	0.020	-0.538	-0.563	0.039	-1.351	-0.237
CACYBP	0.221	-0.583	-0.814	0.005	0.191	0.206
CALCRL	-0.687	-1.154	-0.474	0.339	1.525	-0.074
Calm1 (includes oth	-0.197	1.689	1.679	0.348	2.094	0.336
CAMK2A	0.192	N/A	-2.041	0.146	-3.726	3.846
CAMK2B	0.034	-0.751	-0.801	-0.013	-1.395	0.342
CAMK2D	0.089	-0.828	-0.928	-0.009	-0.891	0.383
CAMK2G	-0.022	0.503	0.514	-0.163	-0.068	0.143
CASP3	-0.442	1.915	2.347	0.261	2.762	0.525
CASP7	-0.038	0.500	0.521	-0.111	-0.017	-0.396
CATSPER2	-0.040	-0.123	-0.079	0.099	-0.299	-0.293
CCL4	0.523	-0.271	-0.806	0.779	0.923	0.181
CCR1	0.117	3.110	2.969	-0.095	-0.735	-0.283
CCR2	0.743	-1.666	-2.424	0.142	-1.205	0.279
CCR3	-0.073	0.535	0.601	-0.262	0.176	-0.288
CCR4	0.138	-2.668	-2.813	-0.074	-1.364	N/A
CCR5	0.303	-0.373	-0.689	0.171	-0.911	-0.047
CCR6	-0.012	-6.444	-6.444	-0.186	-1.693	N/A
CCR7	0.091	-2.898	-3.001	-0.099	-2.038	0.171
CCR9	0.362	-2.255	-2.632	0.138	-1.008	0.355
CCRL2	-0.228	2.637	2.850	0.136	0.805	0.034
CD36	-0.713	0.039	0.744	0.570	2.044	0.192
CDH1	-0.414	-0.068	0.330	-0.581	-0.899	-3.048
CDKN1A	0.428	4.867	4.423	0.119	0.714	-0.077
CELSR1	-0.093	-0.766	-0.684	-0.211	-1.617	-0.257
CELSR2	-0.217	-0.388	-0.174	-0.384	-0.172	0.461
CELSR3	0.255	1.955	1.673	-0.233	-1.955	-0.175
CHRM3	-0.299	2.516	2.800	0.132	-0.869	0.162
CHUK	-0.168	1.196	1.349	0.123	1.804	0.072
CMKLR1	-0.103	2.274	2.358	-0.036	-0.358	-0.556
CNR1	0.621	1.305	0.676	-0.012	-0.201	-0.433
CNR2	-0.065	0.840	0.894	-0.111	0.125	0.062
COPS5	0.094	-0.192	-0.300	0.055	-0.401	-0.032
CREB1	0.035	0.463	0.419	0.197	1.037	-0.094
CREB3	0.061	0.652	0.574	-0.093	-0.296	-0.207
CREB3L1	-0.189	1.624	1.798	0.352	2.485	0.369
CREB3L2	-0.188	0.862	1.033	-0.067	0.736	-0.263
CREB3L3	-2.783	1.876	4.630	-2.303	0.472	-1.859
CREB5	-0.761	2.292	3.031	-0.747	1.827	0.192
CREBBP	-0.455	-0.273	0.174	-0.187	0.430	0.017
CTNNB1	-0.316	1.037	1.340	0.157	1.743	-0.028
CTSD	-0.351	2.281	2.619	0.160	2.396	0.133
CX3CR1	0.218	-1.868	-2.098	-0.038	-0.801	-0.035
CXCR1	N/A	3.834	4.553	N/A	N/A	-0.054
CXCR6	0.312	-0.553	-0.882	-0.250	-0.631	0.598
CYBB	0.238	-0.095	-0.338	-0.060	-0.965	-0.559
CYSLTR1	0.491	1.048	0.525	-0.043	-2.014	-0.881
CYSLTR2	-0.382	2.262	2.634	0.490	2.974	0.379
DLC1	-1.840	1.563	3.367	-2.095	1.116	-0.048
EDNRA	N/A	1.439	1.331	N/A	N/A	1.531
EDNRB	-0.604	4.206	4.769	-0.511	-0.848	-1.070
EGF	0.512	-0.371	-0.907	0.464	-0.105	-0.069
EGFR	-3.056	1.048	4.077	-3.087	-0.186	-1.975
ERBB2	N/A	1.254	1.207	N/A	N/A	0.148
ESR1	-0.574	0.820	1.365	-0.700	-0.858	-1.074
ETV4	N/A	N/A	-0.058	N/A	N/A	N/A
EZR	-0.038	-0.351	-0.323	-0.122	-0.351	0.050
F2R	0.064	1.325	1.252	0.085	1.310	0.535
F2RL1	0.021	-2.839	-2.867	-0.275	-2.524	0.248
F2RL2	-0.479	2.289	2.758	0.397	3.152	0.456
F2RL3	-0.391	1.177	1.548	0.104	2.470	0.635
FCGR1A	0.481	1.376	0.878	0.474	0.293	-0.404
FCGR2A	0.173	2.479	2.289	0.013	0.194	-0.311
FCGR2B	0.084	0.095	-0.004	-0.148	-1.596	-0.500
FCGR3A/FCGR3B	0.192	2.616	2.406	0.334	0.634	-0.245
FFAR1	0.042	-4.930	-4.986	-0.339	-2.491	N/A
FFAR2	0.042	1.885	1.826	N/A	N/A	-0.951
FGFR1	-0.261	3.184	3.433	-0.510	-1.576	-0.782
FGFR2	-2.719	1.797	4.488	-3.096	N/A	-2.996
FGFR3	-1.709	1.728	3.388	-1.993	-0.456	-2.122
FGFR4	-2.442	2.183	4.602	-2.037	N/A	-2.194
FGFRL1	-0.993	1.255	2.221	-0.970	0.782	-0.699
FOS	0.082	1.485	1.391	-0.096	-0.390	-0.016
FPR1	0.200	3.135	2.907	0.023	-0.583	-0.274
FPR2	0.130	3.007	2.853	0.108	0.486	-0.308
FZD1	-0.905	2.479	3.342	N/A	N/A	-0.492
FZD3	N/A	N/A	N/A	N/A	N/A	0.014
FZD4	-1.250	1.701	2.920	-1.440	-0.075	-0.564
FZD5	-0.168	0.495	0.650	-0.015	0.456	-0.068
FZD6	-0.600	0.802	1.386	-1.167	-0.289	0.048
FZD7	-0.295	1.335	1.604	-0.478	-0.169	-0.598
FZD8	-1.288	1.728	2.965	-1.185	N/A	-0.386
GABBR1	0.031	-0.790	-0.833	-0.030	-1.763	0.163
GABBR2	-2.030	2.063	4.054	N/A	N/A	-2.149
GCGR	-2.261	1.984	4.215	-2.047	-0.498	-1.714
GNA11	-0.049	0.226	0.257	-0.373	-0.925	-0.302
GPR107	-0.085	0.374	0.447	-0.040	-0.428	0.022
GPR108	0.101	0.282	0.163	0.001	-0.220	-0.152
GPR132	0.164	-1.232	-1.408	-0.067	-1.488	0.178
GPR137	-0.204	1.182	1.367	-0.176	0.901	0.004
GPR137B	0.124	0.776	0.634	-0.164	-0.408	-0.213
GPR137C	0.130	-1.481	-1.647	0.139	-2.440	0.185
GPR141	0.676	0.628	-0.063	0.425	-0.259	0.019
GPR146	-0.114	1.521	1.618	0.086	1.277	0.151
GPR15	0.033	-3.059	-3.115	-0.108	-1.561	N/A
GPR151	-0.464	-1.320	-0.856	0.057	N/A	N/A
GPR153	N/A	1.550	N/A	N/A	N/A	0.585
GPR155	-0.151	-0.303	-0.164	-0.149	-0.307	-0.543
GPR157	-0.274	0.280	0.555	-0.215	-0.610	1.749
GPR160	0.166	0.324	0.147	0.312	-0.872	-0.104
GPR161	N/A	N/A	-0.126	N/A	N/A	N/A
GPR17	N/A	1.317	4.192	N/A	N/A	-0.701
GPR171	0.250	-2.509	-2.772	0.091	-1.779	0.520
GPR174	0.299	-3.538	-3.850	-0.050	-1.589	0.372
GPR18	0.227	-2.441	-2.682	0.011	-1.578	-0.035
GPR180	-0.140	-0.264	-0.143	-0.270	-0.611	-0.308
GPR182	-0.203	1.917	2.102	-0.205	-2.844	-0.081
GPR183	0.162	-2.970	-3.143	-0.020	-2.039	0.034
GPR19	0.125	0.112	-0.024	0.041	-1.281	-0.100
GPR21	0.156	-0.371	-0.548	0.394	-2.388	0.018
GPR22	-0.226	-1.373	-1.166	-0.147	-4.342	2.301
GPR34	0.159	0.096	-0.085	-0.269	-0.540	-0.215
GPR35	0.482	0.994	0.496	0.072	-0.902	-0.373
GPR4	-0.065	0.065	0.124	-0.278	-2.146	0.045
GPR55	-0.230	0.952	1.171	-0.240	-1.230	0.272
GPR61	N/A	N/A	0.868	N/A	N/A	-0.490
GPR65	0.178	0.574	0.383	0.009	-0.697	0.212
GPR68	0.062	-1.392	-1.461	-0.045	-1.265	0.568
GPR82	0.598	1.964	1.338	0.141	-1.959	-0.404
GPR83	0.202	-0.645	-0.858	0.239	-2.022	0.256
GPR84	N/A	3.044	3.100	N/A	N/A	-0.590
GPR89A/GPR89B	-0.059	-0.328	-0.281	-0.146	-0.846	-0.362
GPRC5B	-0.033	0.729	0.754	-0.149	-0.864	-0.170
GPRC5C	-1.743	2.024	3.721	-2.290	-1.102	-1.845
GRM1	N/A	1.361	1.652	N/A	N/A	0.914
GRM6	0.057	-1.722	-1.809	0.092	-1.368	N/A
GSK3B	-0.226	1.048	1.266	0.201	1.939	0.241
HBEGF	N/A	N/A	N/A	N/A	N/A	1.005
HCAR1	N/A	0.327	N/A	N/A	N/A	-0.410
HCAR2	0.035	2.969	2.906	-0.206	-0.516	-0.144
HIF1A	0.041	-0.218	-0.272	-0.142	-0.827	-0.079
HNF1A	-1.697	0.793	2.444	-1.707	-1.261	-1.514
HRH2	0.162	2.112	1.928	-0.167	-1.230	-0.538
HRH4	0.261	N/A	-2.998	0.373	-0.177	N/A
HTR1B	-0.335	-4.640	-4.285	-0.558	-0.853	N/A
HTR2A	N/A	2.184	2.350	N/A	2.576	0.145
HTR2B	0.166	-0.499	-0.691	0.257	-2.878	-0.508
IGHG1	-0.189	-4.915	-4.802	-0.599	-2.192	N/A
Ighg2b	-0.337	-2.421	-2.085	-0.438	-1.413	-1.740
IGHM	-0.044	-2.571	-2.541	-0.194	-1.301	-0.403
IGKC	0.049	-2.521	-2.583	-0.214	-1.418	-0.421
IKBKB	0.193	-0.898	-1.104	-0.011	-1.388	-0.214
IL10RA	0.260	-0.697	-0.968	-0.013	-1.062	0.188
IL10RB	-0.158	0.693	0.841	0.275	1.554	-0.058
IL18	-0.147	0.399	0.527	-0.438	-0.613	-0.719
IL1B	-0.068	2.962	3.004	-0.300	0.627	-0.447
ILK	-0.400	1.979	2.367	0.323	3.020	0.528
IQGAP1	-0.177	0.679	0.844	-0.283	-0.496	-0.206
IRAK1	-0.388	-0.521	-0.142	0.269	1.190	0.048
IRAK2	0.015	0.100	0.072	-0.132	-0.746	-0.204
IRAK3	-0.004	0.957	0.951	0.011	0.758	0.093
IRAK4	-0.072	1.068	1.124	-0.066	0.405	-0.178
IRF3	0.093	-0.134	-0.240	-0.101	-1.467	-0.270
ITPR1	-0.125	-1.214	-1.097	-0.194	-1.162	-0.458
ITPR2	-0.255	0.441	0.687	-0.099	0.812	0.148
ITPR3	-0.069	-2.274	-2.214	-0.210	-1.562	-0.058
JAK1	-0.217	0.406	0.615	0.008	0.906	0.327
JCHAIN	-0.224	-1.194	-0.981	-0.447	-1.810	-0.234
JUN	0.010	1.372	1.354	-0.232	-0.337	-0.172
KISS1R	0.030	2.379	2.340	-0.015	1.856	0.290
KLK3	1.022	-4.673	-5.403	0.134	0.869	N/A
LEF1	0.124	-2.226	-2.362	-0.079	-2.093	0.243
LGR4	-2.002	1.820	3.778	-2.036	0.788	-1.002
LGR5	-0.095	0.390	0.471	N/A	N/A	-0.600
LPAR1	N/A	2.222	2.797	N/A	N/A	-0.489
LPAR2	-0.098	0.852	0.936	-0.156	-1.111	-0.460
LPAR5	0.114	-0.493	-0.623	-0.108	-1.379	-0.477
LPAR6	0.403	-0.044	-0.463	0.108	-1.718	-0.154
LTB4R	0.211	1.816	1.581	-0.172	0.548	-0.171
LTB4R2	N/A	1.492	2.702	-0.283	3.249	0.797
MAP2K1	0.091	-0.413	-0.516	-0.046	-0.516	-0.203
MAP2K2	-0.631	0.087	0.705	0.311	2.050	0.088
MAP2K3	-0.650	1.410	2.049	0.342	2.691	0.369
MAP2K4	-0.112	0.293	0.392	0.012	0.379	-0.126
MAP2K5	0.065	-0.515	-0.597	-0.002	-0.737	-0.133
MAP2K6	-0.073	-0.589	-0.522	-0.136	-0.289	-0.093
MAP2K7	-0.016	-0.436	-0.430	-0.197	-0.392	0.302
MAP3K8	0.310	0.761	0.431	0.136	-1.425	-0.262
MAPK1	-0.123	1.032	1.144	0.206	1.690	0.091
MAPK11	0.220	-2.846	-3.075	-0.086	-1.550	-0.050
MAPK12	-0.028	-1.514	-1.489	-0.106	-1.875	0.495
MAPK13	0.033	3.280	3.221	-0.231	0.189	-0.439
MAPK14	-0.271	1.336	1.594	-0.051	1.464	0.190
MAPK15	N/A	1.810	3.391	N/A	N/A	-1.881
MAPK3	-0.307	1.716	2.012	0.294	2.457	0.329
MAPK4	N/A	1.406	N/A	N/A	N/A	0.166
MAPK6	-0.398	1.930	2.317	0.394	2.891	0.458
MAPK7	0.140	0.222	0.065	-0.117	-0.536	-0.122
MAPK8	0.023	-0.127	-0.164	-0.049	0.020	-0.174
MAPK9	-0.055	-0.186	-0.142	-0.123	-0.151	-0.255
MCAM	-0.416	0.293	0.695	-0.342	-0.327	0.236
MMP11	0.020	-1.811	-1.824	-0.526	-1.811	-0.038
MMP13	-0.654	0.313	0.973	0.722	2.474	-0.341
MMP14	-1.568	1.334	2.855	-1.355	1.208	-1.321
MMP15	-1.137	1.764	2.859	-1.573	-2.239	-0.443
MMP17	-0.643	-1.800	N/A	-0.903	N/A	N/A
MMP19	-0.632	2.019	2.639	0.238	3.187	0.476
MMP2	-0.928	2.140	3.068	N/A	N/A	0.392
MMP23B	N/A	N/A	N/A	N/A	N/A	0.672
MMP24	-0.190	-1.892	-1.723	-0.182	-1.875	0.161
MMP25	-0.320	2.390	2.690	-0.499	-0.269	-0.424
MMP28	N/A	1.091	1.246	N/A	N/A	0.158
MMP8	-0.205	1.703	1.887	-0.200	0.084	0.238
MMP9	-0.509	2.612	3.107	-0.501	-0.220	-0.322
MYD88	0.080	1.929	1.830	-0.124	0.107	-0.276
MYH9	-0.601	0.918	1.513	-0.265	1.961	0.493
NAPSA	0.158	-1.138	-1.311	0.016	-0.830	-0.153
NCF1	-0.009	0.797	0.795	-0.110	-0.414	-0.119
NCF2	0.068	1.579	1.494	-0.110	-0.402	-0.166
NCL	0.048	-1.030	-1.088	-0.078	-0.539	-0.017
NFKB1	0.078	-0.816	-0.905	-0.100	-0.916	-0.122
NFKB2	-0.006	-0.811	-0.817	-0.170	-0.781	-0.146
NMBR	N/A	N/A	N/A	N/A	N/A	-0.107
NOS2	N/A	N/A	N/A	N/A	N/A	0.610
NOTUM	N/A	1.388	3.253	N/A	N/A	-0.649
NOX4	-2.610	1.381	3.969	-1.851	N/A	-1.559
NR3C1	-0.027	0.521	0.534	-0.194	-0.542	-0.248
NTRK2	-1.836	1.421	3.210	-1.702	0.795	-1.450
OCLN	-1.995	1.378	3.351	-1.918	N/A	-0.980
OPRM1	0.082	0.228	0.123	-0.082	-4.809	0.152
P2RY1	-0.200	2.567	2.756	0.441	2.820	0.403
P2RY10	0.292	-2.340	-2.645	0.093	-1.508	0.307
P2RY12	-0.043	2.570	2.602	0.615	2.651	0.282
P2RY13	0.072	3.378	3.282	-0.156	0.278	-0.302
P2RY14	0.244	0.490	0.237	0.034	-0.489	-0.050
P2RY2	-0.034	2.575	2.587	-0.384	-0.290	-0.297
P2RY4	N/A	1.633	4.390	N/A	N/A	-1.604
P2RY6	0.473	0.455	-0.036	0.175	-0.277	-0.555
PDGFC	N/A	1.431	2.319	N/A	N/A	-1.110
PDIA3	-0.005	0.530	0.517	-0.175	-0.193	-0.480
PIK3C2A	0.044	0.024	-0.034	-0.162	-1.212	-0.157
PIK3C2B	-0.226	-0.361	-0.142	-0.245	0.347	0.274
PIK3C2G	-1.868	1.490	3.320	-1.608	N/A	-1.521
PIK3C3	-0.075	-0.327	-0.260	0.000	0.465	-0.047
PIK3CA	-0.157	0.036	0.182	-0.134	0.295	0.018
PIK3CB	-0.300	1.669	1.955	0.201	1.926	0.049
PIK3CD	0.020	0.302	0.269	-0.157	-1.075	-0.078
PIK3CG	-0.145	0.799	0.933	-0.116	0.264	-0.001
PIK3R1	-0.108	0.488	0.588	0.008	0.754	0.107
PIK3R2	0.021	-0.267	-0.299	-0.220	-1.272	-0.126
PIK3R3	-0.328	0.107	0.416	-0.211	0.020	0.455
PIK3R4	-0.115	-0.538	-0.434	-0.161	-1.749	-0.229
PIK3R5	-0.028	0.935	0.949	-0.112	-0.515	-0.094
PIK3R6	0.377	2.329	1.930	-0.025	-1.003	-0.246
PLA2G10	-0.408	2.805	3.210	0.071	2.991	0.398
PLA2G12A	-0.443	2.042	2.473	0.394	2.898	0.344
PLA2G12B	-2.677	1.597	4.242	-2.034	N/A	-1.832
PLA2G3	0.292	1.049	0.740	0.201	-0.755	-0.187
PLA2G4A	-0.207	2.022	2.218	0.480	2.773	0.368
PLA2G4B	-0.193	-0.964	-0.788	-0.427	-1.377	N/A
PLA2G4C	-0.198	0.745	0.915	0.824	2.257	-0.227
PLA2G6	-0.333	0.798	1.111	-0.121	0.891	-0.580
PLAT	N/A	N/A	N/A	N/A	N/A	-0.035
PLCB1	-0.028	1.955	1.962	0.173	1.819	0.099
PLCB2	0.110	0.535	0.413	-0.178	-1.445	-0.299
PLCB3	0.040	0.787	0.735	-0.186	-0.636	-0.303
PLCB4	-0.374	1.028	1.395	0.015	1.600	0.747
PLCD1	N/A	1.641	1.371	N/A	N/A	0.426
PLCD3	-0.163	0.237	0.388	N/A	N/A	0.720
PLCD4	-0.022	-0.910	-0.889	0.172	-0.787	N/A
PLCE1	-0.716	1.490	2.182	-1.282	-0.810	-0.378
PLCG1	0.048	-1.167	-1.225	-0.066	-1.487	0.093
PLCG2	-0.041	-0.056	-0.025	-0.158	-0.270	-0.050
PLCL1	0.139	-1.315	-1.462	0.053	-0.754	0.233
PLCL2	-0.307	0.191	0.488	0.401	1.752	-0.029
PLG	-2.836	2.155	4.964	-2.725	-0.482	-1.990
PRKACA	-0.412	1.332	1.733	0.009	2.205	0.396
PRKACB	0.021	-0.610	-0.639	-0.059	-0.058	0.075
PRKAG1	-0.059	0.720	0.766	-0.077	0.616	0.064
PRKAG2	0.014	0.161	0.129	-0.109	-0.570	-0.383
PRKAR1A	-0.153	1.331	1.473	0.193	1.901	0.426
PRKAR2A	-0.204	0.440	0.627	-0.199	0.424	-0.078
PRKAR2B	-0.548	1.932	2.469	0.392	2.975	0.466
PRKCA	-0.392	1.549	1.933	-0.023	2.060	0.372
PRKCB	-0.075	-0.963	-0.899	-0.249	-1.024	-0.214
PRKCD	0.034	1.038	0.994	-0.058	0.479	-0.110
PRKCE	-0.227	0.219	0.438	-0.188	0.804	0.354
PRKCG	-0.117	0.018	0.119	-0.312	-1.372	-0.101
PRKCH	0.184	-0.908	-1.103	0.012	-1.081	0.288
PRKCI	-0.072	-0.471	-0.405	-0.318	-0.711	-0.530
PRKCQ	0.015	0.910	0.887	0.172	1.158	0.348
PRKCZ	-0.200	-0.197	-0.011	-0.212	-1.769	-0.843
PRKD1	N/A	N/A	N/A	N/A	N/A	0.478
PRKD3	-0.069	-0.437	-0.384	-0.184	-0.767	-0.408
PROK1	N/A	1.780	3.977	N/A	N/A	-3.664
PROKR1	-0.687	2.019	2.697	0.274	2.886	0.491
PTAFR	-0.145	2.578	2.706	-0.221	-0.105	-0.244
PTGDR2	-0.019	-0.464	-0.450	-0.200	-0.746	-0.226
PTGER1	-0.089	0.729	0.798	-0.237	-1.989	-0.105
PTGER2	0.125	2.387	2.235	-0.194	-1.195	-0.168
PTGER3	-0.002	2.243	2.235	0.231	2.337	-0.245
PTGER4	0.098	-0.780	-0.887	-0.008	-0.966	0.173
PTGIR	0.003	1.881	1.867	0.346	2.142	0.415
PTH1R	-0.132	0.935	1.044	N/A	N/A	-0.115
RAC1	-0.361	1.651	2.000	0.330	2.673	0.345
RAC2	0.044	0.179	0.122	-0.087	-0.209	-0.065
RAC3	-0.205	0.112	0.307	-0.162	0.245	0.507
RAF1	0.030	1.169	1.120	-0.019	0.121	-0.161
RHOA	-0.204	0.449	0.643	0.247	1.315	0.098
ROCK1	-0.122	0.214	0.326	0.214	1.113	-0.050
ROCK2	-0.185	1.375	1.549	0.108	1.904	0.383
RTKN	N/A	1.723	2.979	N/A	N/A	-0.895
RYR2	-0.178	-0.095	0.094	-0.209	-2.532	5.145
S100A1	-0.057	2.193	2.237	0.655	2.808	0.302
S100A10	0.114	1.716	1.591	0.436	2.245	0.200
S100A13	-0.180	0.210	0.379	0.542	1.737	0.247
S100A16	-2.319	1.621	3.914	-1.903	N/A	-0.339
S100A4	0.790	-1.263	-2.072	0.147	-0.406	0.074
S100A6	0.232	1.815	1.558	-0.061	0.378	-0.213
S100A8	-0.348	2.436	2.756	-0.103	1.080	-0.469
S100A9	-0.073	2.729	2.772	-0.151	1.048	-0.197
S100B	N/A	2.546	2.076	N/A	N/A	-1.992
S1PR1	0.106	-1.852	-1.971	-0.130	-1.892	-0.191
S1PR2	0.147	0.952	0.784	-0.109	-0.346	-0.206
S1PR3	-0.304	-1.843	-1.529	-0.421	-1.365	1.158
S1PR4	0.021	-1.068	-1.103	-0.132	-1.391	-0.046
S1PR5	0.192	-0.133	-0.337	0.230	0.074	0.060
SERPINF1	-2.482	1.530	3.983	-2.369	-0.300	-1.473
SERPINF2	-2.691	1.769	4.433	-2.580	-0.209	-1.919
SLC52A2	-0.065	-0.800	-0.744	-0.316	-1.285	-0.213
SMAD1	-0.094	1.325	1.407	0.074	1.196	0.290
SMAD2	0.014	0.220	0.195	0.059	0.475	-0.173
SMAD3	-0.110	0.374	0.472	-0.096	-0.419	-0.120
SMAD4	-0.032	0.638	0.660	0.075	1.041	0.028
SMAD5	-0.015	-0.266	-0.262	-0.091	-0.309	0.086
SMAD6	N/A	1.282	2.537	N/A	N/A	0.418
SMAD7	0.063	-0.899	-0.970	-0.094	-1.920	-0.174
SMAD9	N/A	2.643	3.279	N/A	N/A	-1.417
SMO	-0.368	1.394	1.740	-0.288	1.019	-0.302
SP1	-0.042	0.146	0.179	-0.028	0.035	-0.128
SRC	-0.921	1.853	2.765	0.047	3.230	0.618
SSTR4	0.165	1.281	1.097	0.180	-3.196	0.096
STAT3	-0.183	0.716	0.884	-0.214	-0.132	-0.053
STK38	0.140	0.489	0.335	-0.060	-1.266	-0.218
SUCNR1	N/A	0.981	2.569	N/A	N/A	-1.276
TAS1R3	0.071	0.172	0.087	-0.074	-1.246	0.089
TBK1	0.133	-0.090	-0.235	-0.050	-1.211	-0.197
TBXA2R	-0.027	-1.900	-1.886	-0.245	-1.494	0.021
TCF3	-0.028	-0.920	-0.903	-0.059	0.055	0.041
TCF4	0.023	-1.088	-1.118	-0.152	-0.522	-0.081
Tcf7	0.150	-2.417	-2.578	-0.072	-1.873	0.206
TCF7L1	-0.558	0.831	1.378	N/A	N/A	-0.241
TCF7L2	-0.369	1.140	1.484	-0.254	0.795	-0.478
TGFA	-1.150	1.753	2.867	-1.106	N/A	-1.300
TGFB1	-0.216	0.816	1.022	-0.055	1.454	0.228
TGFB2	-0.591	3.270	3.829	-1.063	-0.567	-0.570
TGFB3	-0.140	-0.543	-0.404	-0.259	-1.048	0.736
TLR4	0.143	2.304	2.141	-0.022	-0.758	-0.306
TNF	0.314	1.190	0.859	-0.026	-0.148	-0.409
TP53	0.013	-1.462	-1.484	-0.025	-0.443	0.064
Tpm1	-0.567	0.636	1.193	0.458	2.344	1.476
Tpm2	-1.192	0.682	1.860	-0.151	3.569	1.234
TPM3	-0.017	0.768	0.775	0.048	0.950	0.350
Tpm4	-0.357	1.743	2.088	0.415	2.847	0.484
TRAF2	-0.024	-0.659	-0.644	-0.145	-0.841	-0.171
TRAF3	-0.079	-0.405	-0.337	-0.168	-0.433	0.058
TRAF6	-0.015	-0.388	-0.383	-0.142	-0.536	-0.175
TRPV5	-0.671	0.506	1.166	0.450	2.591	-0.318
TYK2	0.099	-0.519	-0.631	-0.027	-0.511	0.106
VCAM1	N/A	2.151	3.171	N/A	N/A	-0.348
VEGFA	-1.201	2.270	3.427	-1.186	-0.320	-0.782
VEGFB	-0.199	-0.190	0.008	-0.265	-0.262	1.281
VEGFC	-0.166	0.734	0.881	-0.192	0.964	-0.204
VEGFD	-0.258	1.133	1.378	0.555	2.659	-0.004
VIPR1	0.135	-1.950	-2.093	-0.164	-2.477	-0.239
WNT10A	-0.279	0.182	0.455	-0.417	-2.001	-0.669
WNT2	-0.452	4.344	4.773	N/A	N/A	-0.769
WNT2B	-0.283	0.591	0.848	-0.103	-2.039	-0.203
WNT4	-0.049	1.337	1.375	0.195	-4.195	-0.056
WNT5A	N/A	1.840	5.065	N/A	N/A	-1.391
WNT5B	-0.231	-0.312	-0.101	-0.355	-2.030	-0.737
XCR1	-0.145	2.069	2.198	0.517	2.751	0.575

Supplementary Table S4. Pathogen-induced cytokine storm signaling pathway genes log2 fold change

Genes in the Pathogen-Induced Cytokine Storm Signaling Pathway network	Veh (Day 0) vs Veh (Day 1)	Veh (Day 1) vs Veh+TBI (Day 1)	Veh (Day 1) vs Veh+TBI (Day 1)	Veh (Day 0) vs BIO 300 200 mg/kg (Day 0)	Veh (Day 1) vs BIO 300 200 mg/kg (Day 1)	Veh+TBI (Day 1) vs BIO 300 200 mg/kg (Day 1)
ADRA1A	-1.763	1.252	2.989	N/A	N/A	0.381
AIM2	0.180	-0.878	-1.073	0.068	-0.365	-0.024
AP3B1	-0.100	0.305	0.395	0.004	0.552	-0.096
BHLHE40	-0.468	1.015	1.458	-0.641	0.101	-0.790
C3	-2.401	2.709	5.062	-2.499	-0.467	-2.556
C5	-2.949	1.928	4.849	-2.710	-0.694	-2.162
CASP1	0.382	0.276	-0.117	0.072	-0.902	-0.246
CASP3	-0.442	1.915	2.347	0.261	2.762	0.525
CASP7	-0.038	0.500	0.521	-0.111	-0.017	-0.396
CASP8	0.172	-0.210	-0.395	0.071	-0.122	-0.146
CCL22	-1.066	-1.312	N/A	0.009	2.300	N/A
CCL24	0.473	3.732	3.221	N/A	N/A	-0.912
CCL25	0.149	-0.518	-0.684	0.057	-0.951	-0.156
CCL3L3	-0.165	-1.299	-1.150	0.005	0.599	0.506
CCL4	0.523	-0.271	-0.806	0.779	0.923	0.181
CCL5	0.121	-0.993	-1.126	0.397	1.129	1.046
Ccl6	0.093	1.547	1.443	-0.035	0.302	-0.237
Ccl9	0.340	0.929	0.576	0.053	-0.517	-0.838
CCR1	0.117	3.110	2.969	-0.095	-0.735	-0.283
CCR3	-0.073	0.535	0.601	-0.262	0.176	-0.288
CCR5	0.303	-0.373	-0.689	0.171	-0.911	-0.047
CCR6	-0.012	-6.444	-6.444	-0.186	-1.693	N/A
CD163	-0.076	1.319	1.376	-0.146	0.051	-0.489
CD40LG	0.089	-2.185	-2.291	-0.047	-1.421	0.426
CDH1	-0.414	-0.068	0.330	-0.581	-0.899	-3.048
CERT1	-0.046	0.475	0.508	-0.045	0.143	-0.096
CGAS	0.277	0.203	-0.086	0.153	-0.289	-0.120
CIITA	0.055	-4.730	-4.793	-0.129	-1.784	-0.364
CKLF	0.250	0.547	0.282	0.054	-2.474	-0.285
CLCF1	0.217	-2.237	-2.466	-0.059	-1.934	0.383
CLEC7A	0.251	2.620	2.349	-0.076	-0.162	-0.251
CNTF	-0.112	N/A	-0.137	0.570	1.273	N/A
COL10A1	-0.116	-0.788	-0.688	-0.066	-2.637	0.066
COL11A2	0.205	-0.271	-0.482	-0.070	-1.591	0.015
COL12A1	N/A	N/A	N/A	N/A	N/A	-0.698
COL13A1	N/A	N/A	N/A	N/A	N/A	-1.932
COL15A1	-1.234	1.242	2.443	-1.024	1.485	-0.039
COL16A1	N/A	N/A	N/A	N/A	N/A	-0.377
Col17a1	0.495	1.181	0.642	0.180	-0.678	-0.196
COL18A1	-2.296	2.007	4.274	-2.290	0.316	-1.603
COL1A1	-2.470	1.490	3.946	-3.517	1.810	-1.137
COL1A2	-2.326	1.955	4.259	-2.519	1.970	-0.473
COL20A1	-0.124	0.597	0.708	-0.280	-0.209	-0.022
COL22A1	N/A	1.745	1.679	N/A	N/A	-0.396
COL23A1	0.157	0.283	0.101	0.117	-2.386	-0.190
COL27A1	-0.169	1.161	1.303	-0.476	-1.990	-1.594
COL3A1	-2.069	2.044	4.088	-3.448	0.955	-1.099
COL4A1	-1.464	1.002	2.452	-1.965	-0.537	0.808
COL4A2	-1.161	1.834	2.967	-1.854	0.044	0.330
COL4A3	N/A	N/A	1.240	N/A	N/A	0.878
COL4A4	-0.162	0.211	0.355	-0.443	N/A	0.550
COL5A1	-1.086	0.851	1.929	0.028	2.403	0.107
COL5A2	N/A	2.032	3.816	N/A	N/A	-0.485
COL5A3	-1.652	1.126	2.752	-1.525	N/A	-1.225
COL6A1	N/A	1.500	2.939	N/A	N/A	-0.233
COL6A2	N/A	1.371	3.710	N/A	N/A	-0.398
COL6A3	N/A	0.723	3.360	N/A	N/A	0.107
Col6a4	0.103	-4.188	-4.285	-0.220	-2.581	0.048
COL6A5	N/A	2.632	3.306	N/A	N/A	-1.227
COL6A6	N/A	1.669	2.575	N/A	N/A	-0.107
COL8A1	N/A	N/A	N/A	N/A	N/A	0.538
COL8A2	0.137	N/A	-0.748	0.511	N/A	N/A
COL9A3	0.826	1.145	0.299	N/A	N/A	-0.111
CRP	-3.199	1.427	4.602	-2.417	-0.232	-1.489
CSF2RA	0.124	2.087	1.944	-0.129	-1.036	-0.154
CSF2RB	-0.097	2.045	2.124	-0.250	-0.469	-0.307
CXCL10	-0.419	1.799	2.206	0.194	0.779	-0.270
CXCL12	-3.019	1.784	4.769	-2.471	0.709	-1.095
CXCL13	N/A	3.002	4.232	N/A	N/A	-0.101
CXCL14	N/A	3.145	4.575	N/A	N/A	-0.627
CXCL16	-0.003	0.688	0.676	-0.452	-1.310	-0.248
CXCL2	N/A	2.650	3.973	N/A	N/A	-1.135
Cxcl3	N/A	2.887	2.784	N/A	2.424	0.362
CXCL3	N/A	4.355	4.108	N/A	N/A	-0.160
CXCL6	0.012	2.595	2.562	0.245	2.618	0.095
Cxcl9	-0.387	0.327	0.683	-0.315	0.948	-1.239
CXCR1	N/A	3.834	4.553	N/A	N/A	-0.054
CXCR2	-0.071	3.183	3.230	-0.271	-0.316	-0.235
CXCR3	0.143	-0.056	-0.211	-0.396	-0.563	0.646
CXCR4	0.158	0.405	0.239	0.026	-0.821	-0.082
DHX58	0.211	2.132	1.907	0.100	-0.508	-0.286
Eda	N/A	N/A	N/A	N/A	N/A	0.076
EOMES	-0.019	-1.913	-1.908	-0.049	-0.201	0.902
FADD	0.058	-0.476	-0.549	-0.144	-0.451	0.032
FASLG	0.157	-0.145	-0.319	0.229	0.242	0.805
FGA	-3.418	1.671	5.065	-3.628	-1.248	-2.832
FGB	-3.371	1.388	4.735	-3.671	-0.974	-2.445
FGG	-3.270	1.531	4.777	-3.490	-0.707	-2.533
FOS	0.082	1.485	1.391	-0.096	-0.390	-0.016
FTH1	-0.455	1.246	1.690	0.541	2.586	0.332
FTL	-1.033	1.661	2.688	0.593	4.082	-0.390
GSDMD	0.216	0.911	0.676	-0.024	-1.062	-0.247
GSDME	-0.044	1.341	1.365	-0.130	-0.839	-0.382
HLA-DMA	0.263	-2.895	-3.168	-0.028	-1.036	-0.109
HLA-DMB	0.228	-3.833	-3.843	-0.144	-2.170	-0.293
HLA-DOA	0.232	-3.878	-4.126	-0.079	-1.307	-0.170
HLA-DOB	0.159	-4.463	-4.634	-0.069	-2.076	-0.091
HLA-DQA1	0.133	-3.754	-3.898	-0.085	-1.109	-0.349
HLA-DQB1	0.112	-3.764	-3.886	-0.116	-1.395	-0.303
HLA-DRA	0.243	-3.611	-3.866	-0.024	-1.350	-0.352
HLA-DRB5	0.020	-3.370	-3.401	-0.136	-1.189	-0.327
HMGCR	-0.141	0.473	0.597	-0.104	0.283	-0.089
IFIH1	-0.269	2.250	2.504	0.203	0.760	-0.311
IFNAR1	0.083	0.378	0.282	-0.107	-0.765	-0.227
IFNAR2	-0.170	0.759	0.914	0.243	1.195	-0.199
IFNG	0.718	N/A	-1.124	N/A	0.396	N/A
IFNGR1	0.227	0.131	-0.111	-0.007	-1.188	-0.166
IFNGR2	0.228	0.931	0.687	-0.051	-0.656	-0.064
IL12A	0.256	-1.256	-1.521	-0.038	-0.431	0.026
IL12RB1	-0.285	-0.052	0.218	-0.346	-0.107	0.648
IL12RB2	0.156	-0.274	-0.447	0.237	0.198	0.649
IL15	-0.055	2.509	2.548	0.503	2.071	0.224
IL17C	0.339	1.134	0.779	0.397	-2.880	-0.004
IL18	-0.147	0.399	0.527	-0.438	-0.613	-0.719
IL18R1	0.026	-0.977	-1.016	-0.042	-1.139	0.150
IL18RAP	-0.093	1.433	1.510	-0.043	0.094	-0.042
IL1A	-0.230	1.529	1.741	0.008	1.821	-0.722
IL1B	-0.068	2.962	3.004	-0.300	0.627	-0.447
IL1R1	-1.106	1.155	2.219	-0.505	1.493	-0.513
IL1R2	-0.060	3.718	3.758	0.222	0.503	-0.068
IL1RAP	-0.402	2.521	2.885	-0.675	-0.264	-0.439
IL1RL1	0.019	1.118	1.093	0.524	1.859	-0.080

Genes in the Pathogen-Induced Cytokine Storm Signaling Pathway network	Veh (Day 0) vs Veh (Day 1)	Veh (Day 1) vs Veh+TBI (Day 1)	Veh (Day 1) vs Veh+TBI (Day 1)	Veh (Day 0) vs BIO 300 200 mg/kg (Day 0)	Veh (Day 1) vs BIO 300 200 mg/kg (Day 1)	Veh+TBI (Day 1) vs BIO 300 200 mg/kg (Day 1)
IL1RL2	0.091	-0.418	-0.528	0.160	-0.234	0.244
IL1RN	-0.323	2.614	2.916	-0.212	1.097	-0.164
IL21R	0.041	-0.384	-0.434	-0.006	-0.226	0.478
IL33	N/A	2.091	3.688	N/A	N/A	0.029
IL36G	-0.094	2.470	2.540	-0.311	0.346	-0.105
IL4	-0.051	0.865	0.899	0.190	1.423	0.220
IL6R	0.075	1.025	0.936	-0.143	-1.426	-0.119
IL6ST	-0.119	-0.537	-0.430	-0.278	-2.000	-0.741
IRF1	0.156	0.857	0.685	0.207	-0.623	-0.180
IRF3	0.093	-0.134	-0.240	-0.101	-1.467	-0.270
IRF7	-0.340	2.102	2.431	0.467	1.442	0.045
IRF9	0.128	1.415	1.272	0.268	0.592	-0.087
JAK1	-0.217	0.406	0.615	0.008	0.906	0.327
JAK2	-0.194	1.095	1.280	0.170	1.670	0.160
JUN	0.010	1.372	1.354	-0.232	-0.337	-0.172
LEP	N/A	0.248	N/A	N/A	N/A	0.214
LIF	N/A	N/A	N/A	N/A	N/A	0.200
LTA	0.401	-0.493	-0.922	0.180	-1.174	0.366
LTB	-0.057	-0.147	-0.104	-0.198	-0.636	-0.198
LYST	-0.333	1.535	1.854	-0.316	0.159	-0.150
MAPK1	-0.123	1.032	1.144	0.206	1.690	0.091
MAPK11	0.220	-2.846	-3.075	-0.086	-1.550	-0.050
MAPK12	-0.028	-1.514	-1.489	-0.106	-1.875	0.495
MAPK13	0.033	3.280	3.221	-0.231	0.189	-0.439
MAPK14	-0.271	1.336	1.594	-0.051	1.464	0.190
MAPK15	N/A	1.810	3.391	N/A	N/A	-1.881
MAPK3	-0.307	1.716	2.012	0.294	2.457	0.329
MAPK4	N/A	1.406	N/A	N/A	N/A	0.166
MAPK6	-0.398	1.930	2.317	0.394	2.891	0.458
MAPK7	0.140	0.222	0.065	-0.117	-0.536	-0.122
MAPK8	0.023	-0.127	-0.164	-0.049	0.020	-0.174
MAPK9	-0.055	-0.186	-0.142	-0.123	-0.151	-0.255
MLKL	0.285	1.548	1.235	0.168	0.238	-0.462
MYC	0.096	-1.676	-1.779	-0.057	-1.297	0.109
MYD88	0.080	1.929	1.830	-0.124	0.107	-0.276
Naip1 (includes others)	0.551	2.369	1.935	-0.287	-2.554	-1.117
NFKB1	0.078	-0.816	-0.905	-0.100	-0.916	-0.122
NFKB2	-0.006	-0.811	-0.817	-0.170	-0.781	-0.146
NGFR	-0.112	-2.750	-2.634	-0.445	-1.465	N/A
NLRC3	0.012	-1.954	-1.973	-0.105	-2.043	0.431
NLRC4	0.197	-1.349	-1.559	0.019	-1.684	-0.450
NLRC5	0.101	-0.334	-0.446	0.099	-1.355	0.087
NLRP10	-0.022	-2.005	-1.968	-0.221	-2.071	2.027
NLRP12	-0.180	2.658	2.817	-0.400	-0.358	-0.247
Nlrp1a	0.104	1.477	1.361	0.103	-0.876	-0.125
NLRP3	-0.202	2.687	2.871	-0.229	-0.029	-0.150
NLRP6	-1.271	1.838	3.076	-0.443	3.081	0.037
NOD1	0.074	0.783	0.696	-0.087	-0.533	-0.061
NOD2	0.041	2.043	1.980	0.063	-0.373	-0.043
NOS2	N/A	N/A	N/A	N/A	N/A	0.610
OSM	0.146	0.608	0.434	0.135	-0.194	-0.013
P2RX7	0.147	-0.655	-0.811	-0.069	-1.623	0.191
PDGFC	N/A	1.431	2.319	N/A	N/A	-1.110
PF4	-0.260	2.389	2.637	0.505	3.018	0.173
Ppbp	-0.316	2.179	2.483	0.539	3.032	0.248
PRDM1	0.038	-1.155	-1.209	-0.004	-0.279	0.516
PRF1	0.022	-0.907	-0.942	0.318	0.082	0.726
PROK1	N/A	1.780	3.977	N/A	N/A	-3.664
PYCARD	0.202	0.006	-0.213	0.067	-1.119	-0.231
RAB27A	0.028	1.704	1.661	-0.078	-0.017	-0.197
RIGI	0.006	1.347	1.327	-0.121	-1.186	-0.328
RIPK1	0.137	0.212	0.064	-0.034	-0.254	-0.227
RIPK3	0.182	-0.760	-0.961	0.007	-0.761	-0.419
RORC	-1.815	1.574	3.346	-1.263	-0.127	-1.330
RYR1	-0.495	-1.247	-0.745	-0.938	-0.784	-0.104
RYR2	-0.178	-0.095	0.094	-0.209	-2.532	5.145
RYR3	0.039	-1.520	-1.590	-0.114	-2.291	0.837
SLC2A1	0.080	0.463	0.368	-0.116	-1.034	-0.181
SLC2A2	-2.433	2.087	4.495	-2.399	-0.826	-1.966
SLC2A3	-0.508	2.383	2.881	0.264	2.974	0.432
SLC2A4	-0.605	0.945	1.540	0.579	2.729	0.257
SLC2A5	N/A	1.581	4.169	N/A	N/A	-1.914
SLC2A8	-0.183	0.480	0.650	-0.313	-1.104	-0.252
SOCS3	0.103	2.254	2.123	-0.188	-0.586	-0.197
SPI1	0.034	1.621	1.571	-0.186	0.264	-0.257
STAT1	0.010	0.645	0.624	0.177	-0.483	0.009
STAT3	-0.183	0.716	0.884	-0.214	-0.132	-0.053
STAT4	0.136	-1.408	-1.556	-0.035	-0.991	0.600
STAT5A	-0.037	0.087	0.116	-0.116	-0.382	0.074
STAT5B	-0.163	1.089	1.241	-0.133	0.859	0.216
STING1	0.111	-1.891	-2.014	-0.094	-1.398	0.030
STX11	-0.447	2.170	2.605	0.409	3.058	0.406
STXBP2	0.051	1.053	0.987	0.075	0.927	-0.021
SYK	-0.080	0.524	0.592	-0.087	0.254	0.127
TBK1	0.133	-0.090	-0.235	-0.050	-1.211	-0.197
TBX21	0.027	-0.966	-1.004	0.114	0.503	0.875
TGFB1	-0.216	0.816	1.022	-0.055	1.454	0.228
TGFB2	-0.591	3.270	3.829	-1.063	-0.567	-0.570
TGFB3	-0.140	-0.543	-0.404	-0.259	-1.048	0.736
TICAM1	0.042	-0.175	-0.231	-0.119	-0.737	-0.293
TLR1	0.155	-0.930	-1.098	-0.118	-1.919	-0.315
Tlr11	0.827	-2.354	-3.191	0.823	-1.178	N/A
Tlr12	-0.218	0.379	0.584	-0.181	1.180	0.575
Tlr13	0.064	2.390	2.309	0.067	-0.226	-0.189
TLR2	0.151	1.834	1.660	-0.157	-0.799	-0.131
TLR3	-0.301	1.340	1.610	-0.126	-0.532	-1.006
TLR4	0.143	2.304	2.141	-0.022	-0.758	-0.306
TLR5	-0.398	2.210	2.586	-0.684	-0.927	-0.531
TLR6	0.167	1.649	1.464	-0.006	-1.527	-0.183
TLR7	0.470	-0.066	-0.544	0.128	-0.929	-0.014
TLR8	0.381	1.562	1.164	-0.013	-0.983	-0.123
TLR9	-0.087	-2.828	-2.748	-0.263	-1.970	-0.314
TNF	0.314	1.190	0.859	-0.026	-0.148	-0.409
TNFRSF11B	N/A	N/A	N/A	N/A	N/A	-0.809
TNFRSF1A	0.005	2.184	2.157	-0.124	0.029	-0.206
TNFRSF1B	0.301	1.139	0.824	-0.045	-0.504	-0.204
TNFSF10	0.098	0.224	0.117	0.006	0.012	0.605
TNFSF12	-0.044	0.910	0.932	0.281	1.373	0.038
TNFSF13	0.335	0.921	0.544	N/A	N/A	0.005
TNFSF13B	0.122	3.249	3.097	0.076	-0.170	-0.342
TNFSF14	-0.064	2.359	2.407	0.038	1.297	-0.034
TNFSF4	N/A	4.177	4.565	N/A	N/A	0.308
TNFSF8	0.218	0.945	0.711	0.199	-2.196	0.259
Tnfsf9	-0.025	-0.730	-0.737	-0.124	-0.846	0.778
TRADD	0.206	-0.266	-0.487	0.109	-0.930	-0.208
UNC13D	-0.238	1.153	1.383	0.046	1.720	0.412
VEGFA	-1.201	2.270	3.427	-1.186	-0.320	-0.782
VEGFB	-0.199	-0.190	0.008	-0.265	-0.262	1.281
VEGFC	-0.166	0.734	0.881	-0.192	0.964	-0.204
VEGFD	-0.258	1.133	1.378	0.555	2.659	-0.004
XCL1	0.307	-0.259	-0.573	0.232	0.634	0.592
ZBP1	0.370	1.452	1.068	0.367	-0.695	-0.095
